# A psychological network analysis of parental and peer support on children’s football participation: the bridging role of self-efficacy

**DOI:** 10.3389/fpsyg.2026.1818293

**Published:** 2026-06-17

**Authors:** Xiancheng Zeng, Wei He, Zhigang Guo

**Affiliations:** 1 Football School, Guangzhou Sport University, Guangzhou, China; 2 School of Sport and Health, Guangzhou Sport University, Guangzhou, China

**Keywords:** football participation, gender difference, parental support, peer support, self-efficacy

## Abstract

**Introduction:**

Guided by ecological systems theory, this study employs psychological network analysis to investigate how parental and peer support interact with children’s football participation and whether these ecological dynamics differ by gender.

**Methods:**

We estimated regularized partial correlation networks using EBICglasso on a sample of 287 primary school students (*M* age = 9.704, *SD* = 1.010, age range = 8 to 13 years) and computed centrality, clustering, and bridge centrality metrics. Gender differences were tested via the Network Comparison Test.

**Results:**

Self-efficacy emerged as the most central node and the primary bridge connecting social support to football participation. The global network structure did not differ significantly between boys and girls, suggesting a largely similar psychological architecture across genders. Descriptive patterns suggested that internal persistence items were more central for boys, whereas participation and relational support items were more central for girls.

**Discussion:**

These findings suggest a possible integration of social cognitive and ecological systems perspectives within a network framework. They offer tentative insights that could be considered when developing broadly applicable or gender-responsive interventions to support children’s sustained football participation.

## Introduction

Children’s regular participation in sports represents a critical developmental context that shapes physical health, psychological well-being, and social competencies (Brewer, 2018; Greule et al., 2024). Football offers a unique platform for children to develop these attributes through structured play and social interaction as the world’s most popular team sport (Aguiar et al., 2012; Alesi et al., 2015; Buecker et al., 2021). Despite these established benefits, maintaining children’s football participation remains a challenge in many countries (Schlesinger et al., 2018; Zeng and He, 2024). Moreover, gender gap in football participation reflects broader patterns observed across various physical activities and competitive levels (Chalabaev et al., 2013). Understanding the mechanisms underlying this disparity requires examining how social environments differentially shape boys’ and girls’ football engagement within school curricula.

Existing research has typically examined isolated predictors such as parental support or peer support, leaving it unclear how these factors interact as a system to sustain or reduce gender disparities (Ault et al., 2024; Dorsch et al., 2022). To address these gaps, this study employs psychological network analysis to model the interrelationships among parental support, peer support, self-efficacy, and football participation. By mapping these relationships, this study aims to advance theoretical understanding of children’s sport participation and provide insights for practitioners seeking to bridge the gender gap in football.

### An ecological perspective on children’s sports engagement

Children do not develop their interest in football in isolation but rather their participation emerges from a complex web of social relationships, community resources, and cultural norms that surround them (Agnew et al., 2016). Ecological Systems Theory offers a lens to organize and examine these multi-layered influences (Bronfenbrenner, 1979). In the sports context, multiple environmental levels relate to children’s football participation, including family and peers (Bronfenbrenner and Ceci, 1994).

This multi-level interaction is particularly evident in the context of China’s national school football reform (Qian et al., 2017). Over the past decade, the Chinese government has integrated football into the compulsory education curriculum to promote physical health and social values among children (Zeng and He, 2024). This macro-level policy shift has transformed schools into the primary site for football promotion, moving away from a purely competitive athletic model toward a more inclusive participation model. However, the success of this school-based initiative depends heavily on the surrounding ecological layers. While the government provides facilities and curricula at the exosystem level, the actual engagement of children is still governed by the microsystem, where academic pressure often competes with sports participation for a child’s time. Consequently, understanding how children navigate this environment requires a focused look at how parental approval, peer influence, and personal efficacy beliefs interact within this specific educational framework. Recent research further confirms that cultural factors within the macrosystem are closely linked to the dynamics of the microsystem in youth sport (Cox et al., 2022; Ge et al., 2025). These findings confirm that researchers must examine multiple environmental levels and the interactions between these levels to fully understand sports participation.

### Social support systems of children’s sport

Within the ecological framework, social environments are closely linked to children’s intentions and behaviors toward sports participation (Dowda et al., 2011). Although historical reviews occasionally found weak links between parental influence and physical activity (Sallis et al., 2000), a substantial body of recent evidence confirms that parents play a pivotal role in sustaining children’s engagement (Rhodes et al., 2020). Similarly, peer support has been identified as a key socio-structural determinant of physical activity behavior in children and adolescents, underscoring the importance of social networks for sustained engagement (Wolbring et al., 2025). This influence often varies by demographic and cultural factors. For instance, younger children typically engage in more physical activity because they receive higher levels of parental support (Pugliese and Tinsley, 2007). Gender also mediates this relationship as research indicates that girls are often more sensitive to parental support than boys (Hong et al., 2020). Moreover, parents may provide differential support based on the child’s gender, which sometimes reinforces traditional stereotypes in male-dominated sports like football (Chalabaev et al., 2013). This differential treatment is associated with unequal participation opportunities and relates to the psychological network of children.

As children transition into primary school, their social world undergoes a significant shift from kinship-based relationships to school-based connections (Harris, 1995). While parents remain influential providers of logistics and encouragement, the relative weight of peer influence grows substantially during this developmental stage (Smith, 2003). This transition suggests that children increasingly derive their social validation and behavioral norms from school-mates rather than family members alone.

Consequently, peers represent an essential influence because children spend a majority of their day with friends at school (Brown, 2004). Peer support significantly predicts both the duration of activity and the choice of specific sports (Martínez-Andrés et al., 2017). Recent social network research in physical education settings further demonstrates that students actively select teammates based on shared achievement goals and gender, highlighting the role of homophily and peer influence processes in shaping sport-related social dynamics (Schüßler et al., 2025). Gender differences persist in this domain because girls generally perceive higher levels of support from their peers than boys do (Bokhorst et al., 2010). Additionally, younger children often prioritize peer approval over parental opinions as they seek social belonging (Eccles and Harold, 1991). Positive friendships enhance self-worth and motivation whereas negative experiences such as bullying serve as major barriers to participation (Flores Aguilar et al., 2021; Somerset and Hoare, 2018).

In football specifically, social connections often spark initial interest because most children initiate play because a friend or family member is already involved (Agnew et al., 2016). Persistent verbal persuasion and vicarious experiences from both parents and peers serve as primary sources of a child’s self-efficacy (Chen et al., 2017; Gledhill et al., 2017). While positive social cycles strengthen a child’s belief in their own capabilities, poor social experiences can lead to dropout (Crane and Temple, 2015; Ullrich-French and Smith, 2009).

### Self-efficacy as a proximal psychological process

Within the ecological framework, the impact of the microsystem on child development is often mediated by internal cognitive processes. Among these processes, self-efficacy represents a critical psychological mechanism that links social resources to actual behavior (Bandura, 1997). Self-efficacy refers to an individual’s belief in their capability to organize and execute the actions required to achieve specific goals. In the physical activity domain, these beliefs determine how children approach football by influencing their choice of activities, the effort they expend, and their resilience when facing difficulties (Li et al., 2018).

The integration of self-efficacy into the ecological model explains how parental and peer support effectively translate into sustained football participation. Specifically, self-efficacy is associated with four primary sources within the microsystem. Supportive parents and peers enhance a child’s confidence through verbal persuasion by offering encouragement and vicarious experiences by serving as active role models (Hulteen et al., 2017). Furthermore, stable social networks create the necessary conditions for enactive mastery experiences, allowing children to build competence through successful performance in a supportive environment (Chen et al., 2017).

Crucially, positive social interactions are associated with better regulation of a child’s physiological and affective states (Hu et al., 2021). Research specifically in youth football contexts indicates that high-quality peer relationships and coach-parent support can buffer against performance-related anxiety and negative emotional arousal (Eckardt et al., 2022). When children perceive a mastery-oriented climate within their social circle, their fear of failure diminishes, thereby strengthening their belief in their football capabilities (Martínez-González et al., 2025). Consequently, self-efficacy functions as the vital psychological bridge connecting external environmental supports to a persistent commitment to football.

### Linking environmental layers to the psychological network

The layered structure of the ecological system provides a comprehensive framework for understanding children’s football participation. While the macrosystem encompasses broad cultural norms, the microsystem facilitates the immediate social support that children receive from parents and peers (Cox et al., 2022). This study conceptualizes these environmental influences as a dynamic system where gender norms at the macrosystem level are closely linked to the support mechanisms within the microsystem.

Gender differences in football reflect these environmental influences through several distinct pathways. Cultural expectations often dictate the distribution of sporting resources within the home. For example, households with boys possess a significantly higher density of physical activity equipment compared to those with girls (Sirard et al., 2010). Within the school microsystem, these macro-level norms further influence peer dynamics. Boys often receive more peer encouragement and dominate shared play spaces, while girls may face social exclusion or limited mastery opportunities in football contexts (Riemer and Visio, 2003). These differential social interactions reflect broader assumptions about gender-appropriate sports, creating a psychological landscape where girls must navigate more barriers to build their skill confidence.

However, these multi-layered influences do not operate in a simple linear fashion but instead form a complex web of interactions. Traditional statistical methods often assume unidirectional relationships, yet psychological variables in sports are often mutually reinforcing (Borsboom et al., 2021). For instance, parental encouragement may strengthen a child’s confidence, which then evokes more positive feedback from peers, creating a reciprocal feedback loop. To understand these relationships at a granular level, a psychological network approach is necessary. This method helps explore the core nodes and their interconnections, offering a tentative picture of gender-related psychological patterns in school football (Epskamp et al., 2018).

This research does not attempt to test the full ecological model with its structural and relational dependencies, which would require social network data mapping actual peer relationships and multilevel modeling of classroom effects. Rather, we use the ecological framework heuristically to organize our variables of interest and to conceptualize how children’s subjective perceptions of their microsystems may coalesce into an internalized psychological network. This network represents the functional psychological ecosystem as experienced by the child, not the objective social structure surrounding the child.

### Psychological network analysis as a systemic approach

Psychological variables in youth sport operate as an interconnected system rather than isolated linear predictors (Epskamp and Fried, 2018). Traditional analytic approaches like structural equation modeling (SEM) require predefined directional paths and work well for confirmatory tests, but they cannot easily reveal emergent system properties or identify core bridging variables without prior hypotheses. Psychological network analysis addresses this gap by modeling constructs as nodes and their statistical associations as edges, focusing on direct interrelationships among observed variables without assumptions about latent causes or directional paths (Epskamp et al., 2018).

We use psychological network analysis for three specific reasons that directly address our research questions. First, our study aims to identify the most central nodes in the system of social support, self efficacy, and football participation. Network analysis lets the data reveal these central nodes in a fully data driven way without predefining the core driver of the system before analysis (Burger et al., 2023). Second, our study tests whether self efficacy acts as a bridge between social support and football participation. Traditional mediation analysis focuses on an overall mediating effect at the construct level and generally does not identify which specific items serve this bridging role. Bridge centrality analysis within the network framework answers this question at the item level (Deng et al., 2024). Third, our study tests for gender differences in the entire variable system. Traditional moderation analysis only compares individual path coefficients between groups, whereas the network comparison test allows us to formally compare the global structure and overall connectivity of the psychological system across genders (Van Borkulo et al., 2023).

We also clearly distinguish psychological network analysis from social network analysis to avoid confusion. Social network analysis maps relationships between individuals in a social group, whereas psychological network analysis maps relationships between psychological variables within an individual’s cognitive system (Burger et al., 2023). Our study focuses on each child’s internal psychological system, not the social connections between children. Recent methodological work confirms that cross sectional psychological network analysis is a valid tool for exploratory mapping of psychological systems (Isvoranu and Epskamp, 2023).

Unlike traditional methods such as structural equation modeling, which require pre-specifying mediating pathways or assuming directional relationships, psychological network analysis (PNA) makes no such assumptions and allows the data to reveal the emergent structure of the psychological system—a critical advantage for our exploratory research aims.

### The present study

This study employs psychological network analysis to investigate how parental and peer support interact with children’s football participation through self-efficacy. We have three main objectives: to estimate the network structure and identify central nodes, to examine whether self-efficacy bridges social support to participation, and to test gender differences in network architecture.

Ecological systems theory suggests that children do not internalize social support as a direct input to behavior. Instead the influence of parental and peer support should flow through a proximal psychological process. Self-efficacy theory identifies this process as the individual’s belief in their capability to execute specific actions. We therefore anticipate that self-efficacy beliefs will function as the primary mechanism linking social encouragement to actual football participation. In the context of gender, China’s national school football policy has equalized structural access for boys and girls, yet existing evidence indicates that girls continue to face greater social and motivational barriers in male-dominated sports. We thus expect that the functional organization of self-efficacy relative to social support and participation may differ between genders. Based on these conceptual expectations, we propose the following tentative hypotheses.

*H1*: Self-efficacy is expected to show the highest strength centrality in the network.

*H2*: Self-efficacy may serve as a primary bridge node connecting social support to football participation.

*H3*: We explore whether boys and girls differ in global network structure.

## Methods

### Sample

This study recruited a total of 489 participants between April and May 2025 from a single primary school using a convenience sampling method. We subsequently performed a rigorous data screening process to ensure quality and excluded blank or obviously invalid submissions, resulting in a final analytical sample of 287 children (*M* age = 9.704, *SD* = 1.010, age range 8 to 12 years). The final dataset comprised 128 boys and 125 girls. Although 34 participants did not specify their gender, we retained their data for the overall network estimation to maximize statistical power, while strictly excluding them from the subsequent gender-based Network Comparison Test (NCT). We did not conduct a formal *a priori* power analysis because no established method exists for power calculation in psychological network analysis. However, our sample of 287 exceeds the recommended minimum of 200 for stable network estimation (Epskamp et al., 2018).

### Procedures

This study was approved by the Ethics Committee (2024LLCL-46). Participants were recruited using a convenience sampling approach from a single primary school in Guangzhou between April and May 2025. After obtaining written informed consent from the parents or legal guardians of each participating child, from whom we collected no additional survey or interview data, and receiving administrative authorization from school leadership to conduct the study on campus, research assistants administered paper based questionnaires to students within their regular classrooms. One school principal granted this administrative authorization. We emphasized that participation was entirely voluntary and that all individual responses would remain confidential. Students completed the surveys independently to minimize peer influence. Upon submission of the questionnaires, each participant received a small gift as a token of appreciation.

### Measures

#### Parental support and peer support

We utilized the family and friend support subscales from the Perceived Social Support Scale (MSPSS) to measure the children’s social environment (Zimet et al., 1988). Each subscale contains 4 items and employs a 5-point Likert scale ranging from 1 (very strongly disagree) to 5 (very strongly agree). Regarding parental support, the scale assesses the emotional and instrumental assistance children receive from their family members (e.g., “My parents will help me as much as possible”). In this study, the parental support subscale demonstrated excellent internal consistency and validity (*α* = 0.840, *ω* = 0.842). Similarly, peer support subscale measures the perceived support from friends and classmates (e.g., “My peers will really try to help me”). This subscale also exhibited robust psychometric properties in our sample (α = 0.831, ω = 0.831).

#### Self-efficacy

We assessed children’s self-efficacy for football participation using the Shortened Chinese version of the Physical Activity Self-Efficacy Scale (S-PASESC) (Chen et al., 2019). The instrument consists of 8 items (e.g., “I can do physical exercise on most days of the week.”) and uses a 5-point Likert scale ranging from 1 (not confident at all) to 5 (very confident). In our current study, the scale exhibited excellent internal consistency and construct validity (α = 0.848, ω = 0.849).

#### Football participation

We assessed football participation using two self-report items regarding the children’s engagement in football activities over the previous 7 days. These items measured the frequency and duration of their football play during a typical week (e.g., “Except for the football activities arranged by the school, how many days did you participate in football activities in the past 7 days?”). We identified outliers as cases with z-scores outside the range of −3.29 to +3.29. We then winsorized or removed these cases to avoid undue leverage on partial correlation edges in the psychological network. This standardization process created a continuous participation node that is compatible with the Gaussian Graphical Model (GGM) used in our primary analysis.

#### Demographic variables

We collected basic demographic information from participants, including their self-reported gender (male = 1, female = 2), age, height, and weight. We collected height and weight for descriptive purposes only and did not include them in the network analysis. All measures used in this study, including the full wording of every item in both Chinese and English, are provided in Supplementary Table S1.

### Data analysis

We conducted all statistical analyses using *R* version 4.5.1. To ensure data quality and verify the underlying structures, we first calculated descriptive statistics and Spearman correlations. We evaluated internal consistency using Cronbach’s alpha and McDonald’s omega via the *psych* package.

#### Network estimation and regularization

Our primary analysis involved estimating a regularized partial correlation network to identify the complex relationships among study variables. In this model, nodes represent observed variables and edges represent the partial correlation between two variables after conditioning on all other variables in the network. This approach aligns with the recommended practices for cross-sectional psychological network analysis (Burger et al., 2023). To estimate a parsimonious and interpretable network structure, we employed the graphical Least Absolute Shrinkage and Selection Operator (LASSO) in conjunction with the Extended Bayesian Information Criterion (EBICglasso) (Foygel and Drton, 2010). The LASSO regularization technique shrinks small or spurious edge weights to exactly zero, thereby removing them from the model and limiting the risk of overfitting and false positives. We set the EBIC hyperparameter gamma to 0.1 rather than the default value of 0.5. This choice prioritizes sensitivity over specificity and aligns with the exploratory aims of the present study (Isvoranu and Epskamp, 2023). Preliminary analyses using gamma equal to 0.5 produced an excessively sparse network with very few retained edges, suggesting that a more conservative setting would be overly restrictive for the present data.

#### Linking analytical procedures to hypotheses

We used three analytical procedures to examine our hypotheses. To test H1 that self-efficacy would show the highest strength centrality, we computed strength centrality for each node and compared their values. To test H2 that self-efficacy would serve as the primary bridge node, we computed bridge strength centrality, which quantifies how strongly a node connects to nodes in other theoretical communities. To test H3 exploring whether boys and girls differ in global network structure, we performed the Network Comparison Test (NCT), which compares both the global connectivity level and the pattern of edge weights between genders.

#### Explanation of network indices

Strength centrality measures the sum of absolute edge weights connected to a node, meaning that a node with high strength centrality has many strong connections to other nodes in the network. We used this index to identify the most influential psychological construct within the system, which directly addresses H1. Expected influence is similar to strength centrality but accounts for negative edges, yet because all edges in our network were positive, strength and expected influence produced identical values. We therefore report strength centrality for simplicity and interpretability.

Bridge strength centrality focuses on connections that cross between different theoretical communities. Communities are groups of nodes belonging to the same conceptual domain, for example parental support items or self-efficacy items. Bridge strength measures how strongly a node connects to nodes in other communities. A high bridge strength indicates that the node acts as a connector between different psychological domains. We used bridge strength to test H2, specifically whether self-efficacy items link social support to football participation.

#### Stability and gender comparison

We evaluated the accuracy and stability of the network using the bootnet package (Epskamp et al., 2018). We performed nonparametric bootstrapping with 1,000 iterations to estimate the confidence intervals of edge weights. We used case-dropping bootstrapping to calculate the correlation stability (CS) coefficient. We considered a CS-coefficient above 0.50 as evidence of stable and interpretable centrality.

We examined gender differences using the Network Comparison Test (NCT) with 1,000 permutations (Van Borkulo et al., 2023). This permutation based test allowed us to evaluate two aspects of network invariance across gender. The first aspect is global network strength, a measure of the system‘s overall connectivity. The second aspect is global network structure, a measure of how edge weights are distributed across the entire network. Together these tests address H3.

All analysis scripts are publicly available at Open Science Framework (OSF) (doi: 10.17605/OSF.IO/VH8E5).

## Results

### Descriptive statistics

Descriptive analysis revealed that participants reported relatively high levels of social support, with parental support (*M* = 3.79, *SD* = 1.18) and peer support (*M* = 3.94, *SD* = 1.14) both exceeding the scale midpoint. Self-efficacy scores were also moderate (*M* = 3.51, *SD* = 1.16), while the mean frequency for football participation was 1.81 (*SD* = 1.79). Skewness and kurtosis values for all constructs fell within the range of ± 1.5, confirming that the data satisfied the normality assumptions for network estimation.

**Figure 1:**
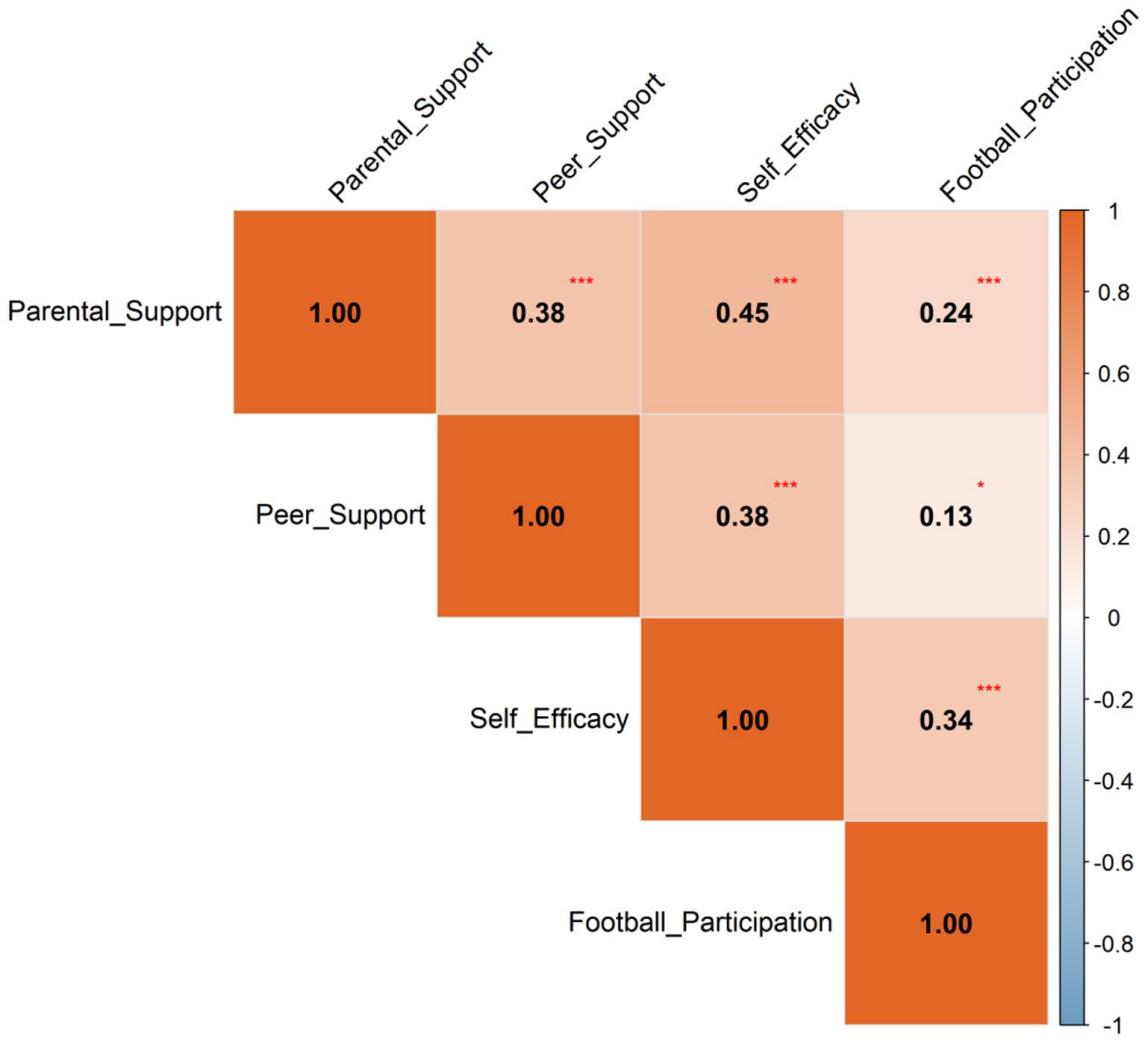
Correlation heatmap. ^*^p < 0.05, ^**^p < 0.01, ^***^p < 0.001.

As illustrated in the correlation heatmap (Figure 1), all study variables were significantly and positively intercorrelated. Self-efficacy exhibited the most robust associations within the network, correlating strongly with parental support (*r* = 0.454, *p* < 0.001) and moderately with peer support (*r* = 0.376, *p* < 0.001). Regarding the outcome variable, football participation was most closely associated with Self-Efficacy (*r* = 0.339, *p* < 0.001), followed by parental support (*r* = 0.241, *p* < 0.001). Although peer support reached statistical significance in its relationship with football participation, the correlation was notably weaker (*r* = 0.128, *p* = 0.031). These results provide the empirical groundwork for using network analysis to identify which specific nodes drive these associations.

### Construct-level network and centrality

**Figure 2:**
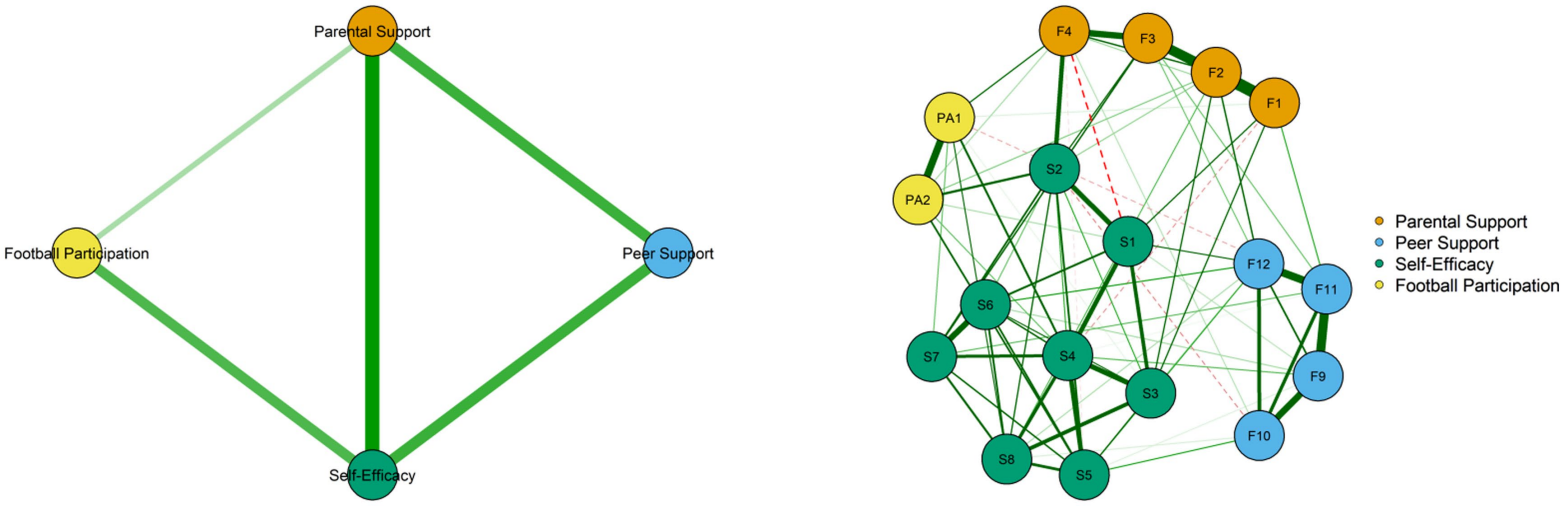
Construct- and item-level network of psychological variables. Edge thickness represents absolute edge weight. F1-F4 = Parental Support items; F9-F12 = Peer Support items; S1-S8 = Self-Efficacy items; PA1-PA2 = Football Participation item.

To address our first objective and test Hypothesis 1, we estimated a regularized partial correlation network at the construct level and computed strength centrality for each node. As shown in Figure 2, the construct-level network comprised four psychological nodes (parental support, peer support, self-efficacy, and football participation). The EBICglasso regularization retained 5 non-zero edges out of 6 possible, yielding a high network density of 83.3%. The average edge weight was 0.229 (*SD* = 0.081), with weights ranging from 0.108 to 0.321. All retained edges were positive, indicating mutually reinforcing relationships among the ecological constructs. The global clustering coefficient was 0.75, demonstrating a substantial degree of local connectivity within the network.

We present this construct-level network primarily as a macro-level visualization of the relationships among the four core constructs. Given the small number of nodes, the centrality metrics derived from this network warrant cautious interpretation (Epskamp and Fried, 2018). The limited degrees of freedom inherent in a four-node network mean that centrality indices can be unstable and may not reliably differentiate the roles of the constructs. Therefore, we report the centrality results in Table 1 for transparency and to provide a general overview, but we base the core findings and substantive interpretations of this study on the subsequent item-level network analysis. The item-level network, comprising 18 nodes, offers a far more granular and robust perspective on the specific psychological mechanisms at play.

**Table 1:** Results construct centrality.

Construct	Strength	Closeness	Betweenness	Expected influence
Self-efficacy	0.791	0.051	2	0.791
Parental support	0.675	0.048	1	0.675
Peer support	0.494	0.042	0	0.494
Football participation	0.330	0.035	0	0.330

Values represent standardized z-scores. Higher values indicate greater centrality.

Construct centrality analysis, summarized in Table 1, identified self-efficacy as the most influential construct with a strength centrality of 0.791. This was followed by parental support (0.675), peer support (0.494), and football participation (0.330). Self-efficacy also exhibited the highest bridge strength, confirming its pivotal role in connecting the social support system with behavioral engagement. In this network, strength and expected influence values were identical for all nodes because all associations were positive.

Self-efficacy was most strongly connected to parental support (weight = 0.321), followed by peer support (weight = 0.248) and football participation (weight = 0.222). The robust edge between parental support and self-efficacy, coupled with the relatively weaker direct link from parental support to football participation (weight = 0.108), suggests that parental influence on children’s football involvement is primarily manifested through the enhancement of their perceived competence. Peer support showed moderate connections to both self-efficacy (0.248) and parental support (0.246), but was the most distal construct relative to actual football participation.

We assessed the robustness of the construct-level network using a case-dropping bootstrap (1,000 iterations). The correlation stability (CS) coefficients for strength and expected influence were both 0.596, exceeding the preferred 0.50 threshold. This confirms that node rankings remain highly stable even with a 59.6% reduction in the sample size. Additionally, CS-coefficients for closeness (0.439) and betweenness (0.282) surpassed the 0.25 requirement for reliable interpretation.

### Item-level network analysis

To address our second objective and test Hypothesis 2, we estimated the item level network and computed bridge centrality metrics to identify which nodes connect social support to football participation. We built an 18-item item-level network, which revealed more granular psychological interactions between our study variables. EBICglasso regularization kept 43.3% of all possible edges to maintain a parsimonious network structure. Nearly all edges were positive, a pattern that indicated a mutually reinforcing system of associations. A global clustering coefficient of 0.475 showed robust local interdependence among the specific behaviors and perceptions we measured.

Examination of edge weights, supported by a non-parametric bootstrap procedure (1,000 iterations), confirmed the precision of our estimates. The strongest connection was observed between F1 (My parents will help me as much as possible) and F2 (I get the emotional help and support I need from my parents) (weight = 0.560), followed by F2 and F3 (I can talk about my problems with my parents) (0.444). Within the self-efficacy domain, S2 (I can ask my parents or other adults to do physical exercise with me) and S1 (I can do physical exercise on most days of the week) (0.236) showed robust connectivity. Notably, a significant cross-domain edge was identified between F4 (My parents are willing to help me make decisions) and S2 (weight = 0.208). The relatively narrow 95% confidence intervals (CIs) obtained from the bootstrap results indicate that these edge weights were estimated with high accuracy, representing reliable associations rather than sampling noise.

Community detection using the Louvain algorithm perfectly recovered the four theoretical constructs (Adjusted Rand Index, ARI = 1.00; Modularity = 0.171). This perfect alignment between empirical data and the theoretical framework provides strong evidence for the structural validity of our scales. The recovery of the four predefined communities primarily reflects the internal consistency of the scales. The unique contribution of this network analysis, however, lies in identifying cross-community edges that connect items from different theoretical constructs. These bridge edges represent functional couplings between distinct psychological domains. For example, a notable bridge edge connected F4 (My parents are willing to help me make decisions) to S2 (I can ask my parents or other adults to do physical exercise with me) with a weight of 0.208. Traditional methods such as factor analysis or SEM typically focus on relationships among latent constructs and cannot readily identify such fine-grained cross-domain associations. The following bridge centrality analysis systematically examines these connections to pinpoint the specific items that link the social microsystem to individual behavior.

**Figure 3:**
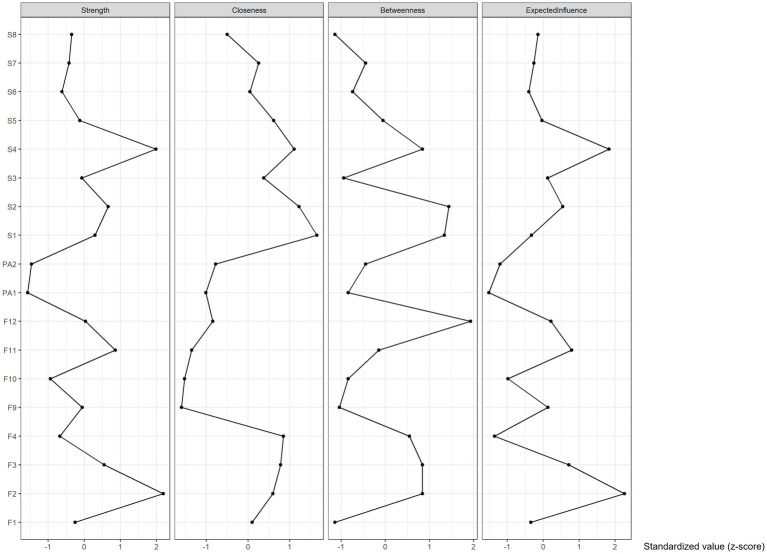
Item-level centrality plot (strength, closeness, and expected influence). F1-F4 = Parental Support items; F9-F12 = Peer Support items; S1-S8 = Self-Efficacy items; PA1-PA2 = Football Participation items.

Centrality analysis (Table 2 and Figure 3) identified F2 and S2 as the most influential nodes. The stability of these findings was rigorously evaluated using the case-dropping bootstrap. The CS-coefficients for Expected Influence (CS = 0.596) and Strength (CS = 0.516) both exceeded the preferred threshold of 0.50, indicating excellent stability for these primary metrics. Closeness centrality (CS = 0.282) also surpassed the recommended minimum of 0.25, suggesting acceptable stability. In contrast, betweenness centrality demonstrated zero stability (CS < 0.01), aligning with prior evidence that betweenness is highly unstable in networks of this size (Borsboom et al., 2021; Epskamp et al., 2018). Consequently, while betweenness is reported in Table 2 for transparency, we refrain from its psychological interpretation.

**Table 2:** Results item centrality.

Item	Strength	Closeness	Betweenness	Expected influence
F1	0.858	0.003	0	0.803
F2	1.299	0.004	40	1.299
F3	1.004	0.004	40	1.004
F4	0.781	0.004	34	0.609
F9	0.893	0.003	2	0.893
F10	0.734	0.003	6	0.681
F11	1.059	0.003	20	1.019
F12	0.91	0.003	62	0.910
S1	0.958	0.004	50	0.805
S2	1.024	0.004	52	0.971
S3	0.891	0.004	4	0.891
S4	1.263	0.004	40	1.217
S5	0.881	0.004	22	0.862
S6	0.792	0.003	8	0.792
S7	0.828	0.004	14	0.819
S8	0.840	0.003	0	0.840
PA1	0.620	0.003	6	0.580
PA2	0.639	0.003	14	0.639

Values represent standardized *z*-scores. Higher values indicate greater centrality. Due to low stability (CS < 0.01), betweenness centrality at the item level is reported for transparency but not interpreted in the main text. F1-F4 = Parental Support items; F9-F12 = Peer Support items; S1-S8 = Self-Efficacy items; PA1-PA2 = Football Participation items. For the full wording of all items, please refer to Supplementary Table S1.

**Figure 4:**
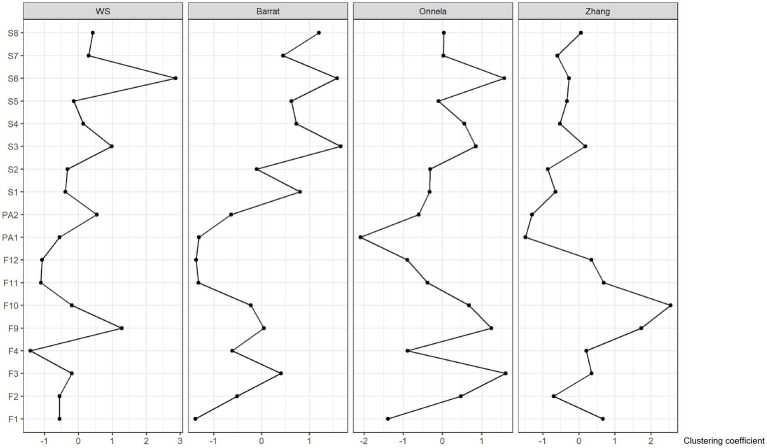
Item-level clustering plot (WS, Barrat, Onnela, Zhang). F1-F4 = Parental Support items; F9-F12 = Peer Support items; S1-S8 = Self-Efficacy items; PA1-PA2 = Football Participation item. For the full wording of all items, please refer to Supplementary Table S1.

Local connectivity was assessed using four clustering algorithms (Table 3 and Figure 4). S3 (I can do physical exercise in my spare time on most days of the week even if I can watch TV or play video games instead) showed the highest Barrat-weighted coefficient (0.695), suggesting that self-efficacy items form the most cohesive functional unit. Collectively, the high community recovery (ARI = 1.00) and the robust stability of centrality and edge weights provide a solid foundation for the subsequent group comparison analysis.

**Table 3:** Clustering coefficients of items.

Node	WS unweighted	Barrat weighted	Onnela weighted	Zhang weighted
F1	0.500	0.414	0.059	0.188
F2	0.500	0.495	0.091	0.209
F3	0.524	0.580	0.105	0.234
F4	0.444	0.485	0.076	0.173
F9	0.619	0.547	0.084	0.238
F10	0.524	0.521	0.09	0.215
F11	0.464	0.419	0.082	0.216
F12	0.467	0.415	0.072	0.216
S1	0.511	0.616	0.076	0.222
S2	0.515	0.532	0.075	0.271
S3	0.600	0.695	0.081	0.310
S4	0.545	0.609	0.084	0.248
S5	0.527	0.600	0.077	0.281
S6	0.722	0.688	0.076	0.393
S7	0.556	0.584	0.075	0.311
S8	0.564	0.653	0.074	0.286
PA1	0.500	0.421	0.047	0.201
PA2	0.571	0.483	0.063	0.228

F1-F4 = Parental Support items; F9-F12 = Peer Support items; S1-S8 = Self-Efficacy items; PA1-PA2 = Football Participation item. For the full wording of all items, please refer to Supplementary Table S1.

### Network comparison test and descriptive patterns by gender

**Figure 5:**
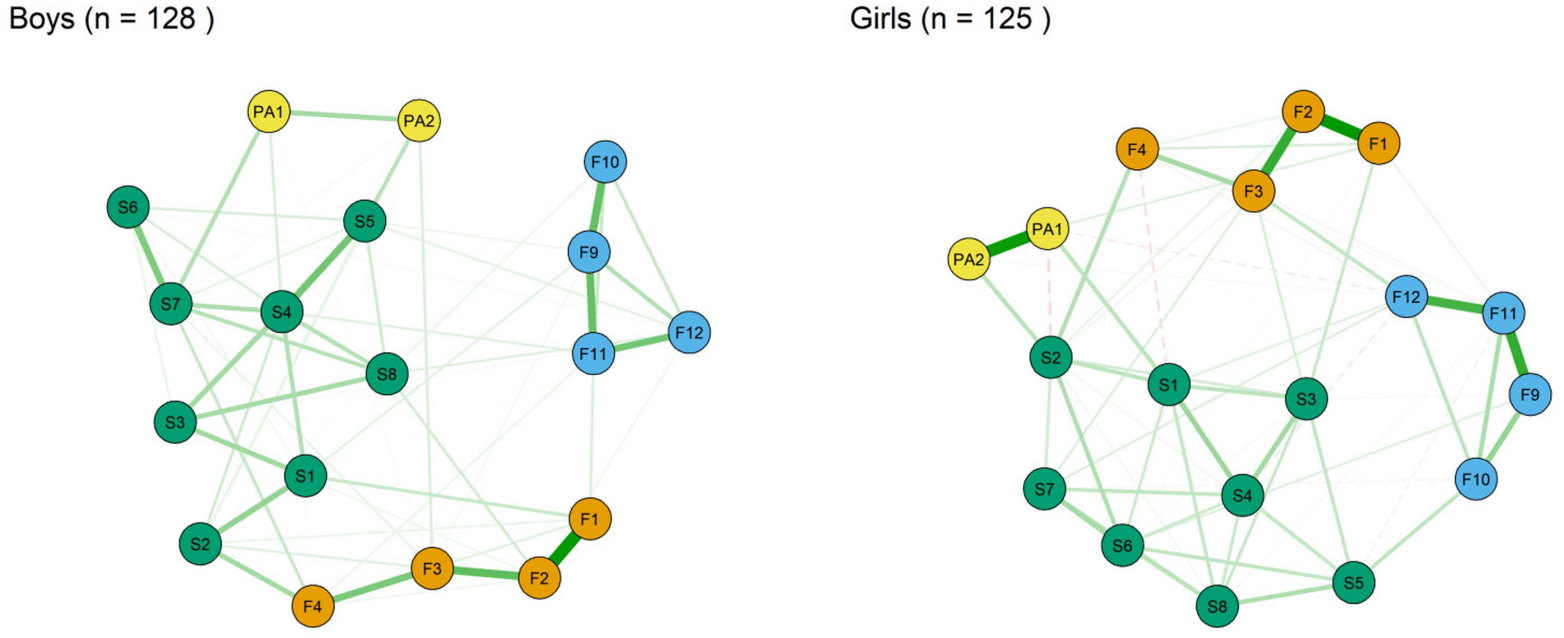
Network structures by gender. F1-F4 = Parental Support items; F9-F12 = Peer Support items; S1-S8 = Self-Efficacy items; PA1-PA2 = Football Participation items. Edge thickness represents absolute edge weight.

To address our third objective and test Hypothesis 3, we conducted a Network Comparison Test (NCT) with 1,000 permutations to compare global network structure and strength across genders. This analysis assessed differences in global network strength and overall structure between the male (*n* = 128) and female (*n* = 125) networks (as shown in Figure 5).

The results indicated that the overall architecture of the football participation network is highly consistent across genders. We found no statistically significant difference in global strength invariance (*S_obs_* = 0.02, *p* = 0.658), suggesting that the total level of connectivity within the system is similar for both boys and girls. Furthermore, the test for network structure invariance revealed no significant divergence in the distribution of edge weights (*M_obs_* = 0.18, *p* = 0.958). These results suggest that the core psychological architecture is largely similar across genders, indicating that the fundamental interplay between social support and self-efficacy may operate similarly for boys and girls.

Although the global structure remained invariant, we examined node level metrics descriptively. Table 4 shows the strength centrality values for each item by gender. In the male network, item S4 showed the highest strength centrality (z = 1.398) and its value was higher than in the female network (z = 1.008). In the female network, item PA1 showed a higher centrality (z = 0.931) compared to the male network (z = 0.477), and item S6 also showed a higher centrality in girls (z = 0.955) than in boys (z = 0.553). We emphasize that these node level comparisons are purely descriptive. We did not perform formal statistical tests on individual node centrality differences because such comparisons are not supported by established inferential procedures in network psychometrics. Therefore we interpret these patterns only as exploratory observations that may inform future hypothesis generation, not as evidence of gender differences.

**Table 4:** Descriptive comparison of items with largest centrality differences between genders.

Item	Construct	Boys strength	Girls strength	Strength difference	Strength difference direction
PA1	Football participation	0.477	0.931	−0.454	Higher in Girls
S6	Self-efficacy	0.553	0.955	−0.402	Higher in Girls
S4	Self-efficacy	1.398	1.008	0.390	Higher in Boys
S2	Self-efficacy	0.775	1.111	−0.337	Higher in Girls
F11	Peer support	0.989	1.295	−0.307	Higher in Girls

F1-F4 = Parental Support items; F9-F12 = Peer Support items; S1-S8 = Self-Efficacy items; PA1-PA2 = Football Participation items. Strength values represent standardized *z*-scores. Positive differences indicate higher centrality in boys. These values are descriptive only. We did not perform statistical tests on node level centrality differences. Only the top 5 items with largest absolute differences are shown. The full wording of all items is provided in Supplementary Table S1.

Conversely, for girls, PA1 occupied a much more central position (*z* = 0.931) compared to boys (*z* = 0.477), with a notable strength difference of 0.454. Similarly, S6 exhibited higher centrality in girls (*z* = 0.955) than in boys (*z* = 0.553). Descriptively, these patterns suggest that for female participants actual participation behaviors and specific efficacy beliefs may be more tightly connected within the psychological system, whereas for males these behaviors may be less centrally embedded. However, we interpret these observations with caution as they are purely descriptive and not based on formal statistical tests.

Bridge centrality analysis provided a descriptive comparison of the nodes connecting different psychological constructs across genders (Table 5). Item S2 served as the primary bridge node for both groups, though its bridge strength was descriptively higher in the female network (BS = 0.511) than in the male network (BS = 0.388). Secondary pathways also showed descriptive differences. In the male network, F4 (parental decision-making support) emerged as a notable bridge (BS = 0.384), whereas in the female network F12 (peer support, BS = 0.393) and F3 (parental communication, BS = 0.373) appeared as additional connectors. We note that these bridge strength differences did not undergo formal statistical testing and therefore represent descriptive observations. Future research with larger samples may examine whether these patterns reflect genuine gender-specific bridging mechanisms.

**Table 5:** Descriptive comparison of top bridge items by gender group.

Rank	Boys	Bridge strength	Girls	Bridge strength
1	S2 (Self-efficacy)	0.388	S2 (Self-efficacy)	0.511
2	F4 (Parental support)	0.384	F12 (Peer support)	0.393
3	S7 (Self-efficacy)	0.366	F3 (Parental support)	0.373

Bridge strength values represent the item’s importance in connecting different theoretical constructs within the network. These values are descriptive only. We did not perform statistical tests on bridge strength differences. For the full wording of all items, please refer to Supplementary Table S1. F1-F4 = Parental Support items; F9-F12 = Peer Support items; S1-S8 = Self-Efficacy items; PA1-PA2 = Football Participation items.

Thus, the NCT revealed no significant differences in global network structure or global strength between boys and girls. These results support the notion that the core psychological architecture linking social support, self-efficacy, and football participation is largely gender-invariant. Descriptive patterns at the node level suggested that certain items, such as S4 for boys and PA1 for girls, may play relatively more central roles in their respective networks. However, these descriptive observations require confirmation in future studies with larger samples. We therefore refrain from drawing strong conclusions about gender-specific mechanisms and instead offer these patterns as preliminary insights that may inform hypothesis generation for subsequent research.

## Discussion

The present study explored children‘s football participation as an interconnected psychological system. Self-efficacy consistently showed the highest centrality and served as the primary bridge connecting social support to participation, which descriptively supports H1 and H2. The global network architecture did not differ significantly between boys and girls, suggesting a largely similar structure across genders (H3). Descriptive node-level patterns suggested some divergence, with internal persistence items appearing more central for boys and participation and relational support items more central for girls. We emphasize that these node-level observations are purely descriptive and not based on formal statistical tests.

### Self-efficacy as the central hub in the psychological network

Self-efficacy emerged as the most central node in the network, supporting that efficacy beliefs function as the primary mechanism linking environmental factors to behavior (Bandura, 1997). In our network, self-efficacy showed the strongest connections to both parental and peer support, while also maintaining a direct link to football participation.

The strong connection between parental support and self-efficacy deserves attention. Parents who offer encouragement and positive feedback help children develop confidence in their football abilities (Chek and Pandey, 2016; Stake, 2006). This finding fits with Self-efficacy Theory about the sources of self-efficacy, especially verbal persuasion and enactive mastery experiences (Bandura, 1997). The slightly weaker link between peer support and self-efficacy may reflect developmental timing, as peer influence typically grows during adolescence (Harris, 1995).

The direct connection from parental support to football participation was relatively weak (weight = 0.108). However, the indirect pathway through self-efficacy was much stronger. This pattern suggests that parental support shapes children’s football participation mainly by building their perceived competence, not through direct pressure. This interpretation aligns with self-determination theory, which emphasizes that autonomy support leads to lasting engagement (Ryan and Deci, 2000).

At the item level, the connection between F4 (My parents are willing to help me make decisions) and S2 (I can ask my parents or other adults to do physical exercise with me) was particularly notable (weight = 0.208). This cross-domain edge suggests that when parents help children make decisions about their activities, children become more confident in mobilizing adult support for physical activity.

The centrality ranking also revealed an important developmental pattern. Parental support (strength = 0.675) emerged as the second most influential node, substantially ahead of peer support (strength = 0.494). This finding reflects the developmental stage of our sample. At ages 8 to 12, parents remain the primary source of social support and guidance, while peer influence is still emerging. Parents provide not only logistical support for football participation but also the verbal encouragement and feedback that shape children’s self-efficacy beliefs.

However, peer support should not be overlooked. Although it ranked third, its connections to both parental support and self-efficacy were meaningful (weights = 0.246 and 0.248). This suggests that peers and parents are not competing influences but work together. When parents support a child’s football involvement, they may also facilitate peer interactions by arranging playdates or encouraging team participation. The moderate connection between peer support and football participation (*r* = 0.128) also suggests that peer influence on actual behavior may strengthen as children move into adolescence, consistent with developmental trends (Innes et al., 2020).

These patterns highlight the importance of considering both social supports in intervention design (Bengtsson et al., 2024). Programs should leverage parental involvement while also creating conditions for positive peer interactions, with parents laying the foundation and peers providing the social context that sustains engagement.

### Descriptive patterns in node level centrality across genders

For H3, although the global network structure did not differ between boys and girls, node-level centrality showed descriptive patterns that differed by gender. These differences show how macrosystem influences, such as cultural gender norms, shape functioning at the microsystem level.

The most notable descriptive difference appeared for item S4 (I can do physical exercise on most days of the week even if it is very hot or cold outside), which measures confidence in persisting despite environmental barriers such as hot or cold weather. This item showed higher centrality in the male network (z = 1.398) than in the female network (z = 1.008). Descriptively, this pattern aligns with the notion that for boys confidence in persisting through external challenges may be particularly salient within the psychological ecosystem. This observation resonates with prior work on gender socialization linking masculinity with perseverance (Knowlton and Newland, 2024; Leaper and Friedman, 2007). In the female network, items related to actual participation behavior (PA1) and specific efficacy beliefs (S2, S6) exhibited descriptively higher centrality. These patterns suggest that for girls the act of participating and the ability to overcome social and environmental barriers may be especially relevant, a notion consistent with prior findings (Zhou and Liu, 2025). We emphasize that these node level comparisons are purely descriptive and did not involve formal hypothesis testing. Therefore we present these patterns only as exploratory observations that may inform future research, not as evidence of statistically reliable gender differences.

These findings must be interpreted within the context of China’s national school football policy. Over the past decade, this policy has integrated football into the compulsory curriculum, creating formally equal opportunities for boys and girls to participate (Qian et al., 2017; Zeng and He, 2024). Despite this policy driven equality at the structural level, the results reveal persistent gender differences at the psychological level. This pattern suggests that equal opportunity does not automatically translate into equal psychological experience. The policy has successfully changed the exosystem, but changes at the microsystem and individual levels may lag behind.

Bridge centrality analysis provided a descriptive comparison of cross-community connections. Item S2 served as the primary bridge node for both genders, though its bridge strength was descriptively higher in the female network (0.511) than in the male network (0.388). This pattern suggests that confidence in mobilizing adult support may be a particularly important connector for girls, a notion consistent with the idea that girls in male-dominated activities may rely more heavily on support-seeking efficacy (Beets et al., 2007; Wang et al., 2025). Secondary bridge pathways also showed descriptive differences. In the male network, F4 (My parents are willing to help me make decisions.) emerged as a notable bridge, whereas in the female network F12 (I can talk about my problems with my peers.) and F3 (I can talk about my problems with my parents.) appeared as additional connectors. These descriptive observations align with research on gender differences in social orientation, which suggests that girls place greater emphasis on relational connectedness while boys focus more on instrumental support (Schüßler and Fuhse, 2025). We reiterate that these bridge strength differences are descriptive and require formal statistical confirmation in future studies.

This divergence aligns with research on gender differences in social orientation, which shows that girls place greater emphasis on relational connectedness while boys focus more on instrumental support. In the football context, these general tendencies manifest as different pathways through which social support facilitates participation.

### Practical implications

Our findings point to several concrete actions for different stakeholders. At the policy level, the persistent gender differences in our network carry a clear message. Equal access to facilities, while necessary, is not sufficient. Teacher training programs focused on motivational climates and gender sensitive coaching would complement existing infrastructure investments.

Schools implementing the national football policy should consider that building self-efficacy requires deliberate effort alongside skill development. Mastery oriented training with positive feedback can achieve this.

Parents appear to play a crucial role, though the pattern of associations differs from what many might assume. The weaker direct edge between parental support and participation, contrasted with the stronger connection through self-efficacy, suggests that confidence building is more closely associated with participation than direct pressure.

### Limitations and future studies

Several limitations warrant consideration in this study. Firstly, the cross-sectional design prevents causal inference about relationships among variables. Longitudinal research tracking children’s networks over time could illuminate temporal dynamics and directional influences (Zhang et al., 2023).

Secondly, our sample was drawn from a single primary school in Guangzhou, China, limiting generalizability. Cross-cultural research comparing networks across societies with different gender norms and football cultures could reveal how macrosystem variation shapes network architecture.

Additionally, the present study operationalized Bronfenbrenner’s ecological systems at the level of individual perceptions, which limits our capacity to model structural interdependencies such as peer norms and classroom-level dynamics. We assessed how children subjectively experience their microsystems, not the objective properties of those microsystems. Future research could combine psychological network analysis with social network analysis to examine how objectively measured social structures relate to individually perceived psychological ecosystems.

Finally, our measurement relied on self-report questionnaires, which may be subject to social desirability bias. Future research should incorporate multiple data sources including parent reports, coach ratings, and objective participation measures.

## Conclusion

This study identified self-efficacy as the central hub connecting social support to children’s football participation. Although the global network structure was consistent across genders, descriptive patterns suggested different pathways through which support connects to participation. These findings integrate social cognitive and ecological systems perspectives within a network framework and provide initial exploratory insights that could inform the development of broadly applicable or gender-responsive interventions to promote children’s sustained football engagement.

## Data Availability

The raw data supporting the conclusions of this article will be made available by the authors, without undue reservation.
