# Supplementary material for: A psychological network analysis of parental and peer support on children’s football participation: the bridging role of self-efficacy
Source: Front Psychol. 2026 Jun 17;17:1818293. doi: 10.3389/fpsyg.2026.1818293 (PMC13319038; doi:10.3389/fpsyg.2026.1818293)
Supplement: Supplementary file 1 [file Supplementary_file_1.DOCX]

# Supplementary Table S1. Full Item Wording for All Scales (Chinese and English)

## Scale 1: Perceived Social Support Scale/感知社会支持量表

**中文备注**：本量表聚焦体育背景下的社会支持，针对父母及他人对您运动相关的支持情况，请选择你认为最合适的选项（打钩“√”），选项包括：非常不同意、不太同意、一般、比较同意、非常同意

**English Note**：This scale focuses on social support in the sports context. For the support from parents and others related to your sports activities, please select the most appropriate option (tick "√"). The options include: Strongly Disagree, Disagree, Neutral, Agree, Strongly Agree

| 序号 | Chinese | English |
| --- | --- | --- |
| 1 | 我的父母会尽可能的帮助我。 | My parents will help me as much as possible. |
| 2 | 我从父母那里得到了我需要的情感帮助和支持。 | I get the emotional help and support I need from my parents. |
| 3 | 我可以和我的父母讨论我遇到的问题。 | I can talk about my problems with my parents. |
| 4 | 我的父母愿意帮助我做出决定。 | My parents are willing to help me make decisions. |
| 5 | 在我遇到问题时有些人(老师、同伴、父母)会出现在我的身旁。 | There are people (teachers, peers, parents) who are there for me when I am in trouble. |
| 6 | 我能够与有些人(老师、同伴、父母)共享快乐与忧伤。 | I can share my joys and sorrows with people (teachers, peers, parents). |
| 7 | 当我有困难时有些人(老师、同伴、父母)是安慰我的真正源泉。 | There are people (teachers, peers, parents) who are a real source of comfort to me when I am in difficulty. |
| 8 | 在我的生活中有某些人(老师、同伴、父母)关心着我的感情。 | There are people (teachers, peers, parents) who care about my feelings in my life. |
| 9 | 我的同伴们能真正的帮助我。 | My peers will really try to help me. |
| 10 | 在发生困难时我可以依靠我的同伴们。 | I can count on my peers when things go wrong. |
| 11 | 我的同伴们能与我分享快乐与忧伤。 | My peers can share my joys and sorrows with me. |
| 12 | 我能与同伴们讨论自己的难题。 | I can talk about my problems with my peers. |

## Scale 2: Football Physical Activity Questionnaire/足球运动行为调查问卷

**中文备注**：请根据自身实际情况如实填写具体数字

**English Note**：Please fill in the specific numbers truthfully according to your actual situation

| 序号 | Chinese | English |
| --- | --- | --- |
| 1 | 除了学校安排的足球运动，在过去7天内，你大概有几天参与足球运动：_____天（请填0、1、2、3、4、5、6、7）。 | Except for the football activities arranged by the school, how many days did you participate in football activities in the past 7 days? ______ days (Please fill in 0,1,2,3,4,5,6,7) |
| 2 | 除了学校安排的足球运动，在过去7天内参与足球运动中，你每次足球运动的时长大约是：_____分钟（如没有请填“0”）。 | Except for the football activities arranged by the school, what was the approximate duration of each football activity you participated in over the past 7 days? ______ minutes (Please fill in 0 if none) |

## Scale 3: Shortened Physical Activity Self-Efficacy Scale (Chinese Version)/简版体育活动自我效能感量表（中文版）

**中文备注**：本量表聚焦体育锻炼相关自我效能感，请选择你认为最合适的选项（打钩“√”），选项包括：非常不同意、不太同意、一般、比较同意、非常同意

**English Note**：This scale focuses on self-efficacy related to physical exercise. Please select the most appropriate option (tick "√"). The options include: Strongly Disagree, Disagree, Neutral, Agree, Strongly Agree

| 序号 | Chinese | English |
| --- | --- | --- |
| 1 | 在每周大部分天数中，我会锻炼身体。 | I can do physical exercise on most days of the week. |
| 2 | 我可以要求父母或其他成年人和我一起锻炼身体。 | I can ask my parents or other adults to do physical exercise with me. |
| 3 | 即使我可以看电视或玩电子游戏，我仍然可以在每周大部分天数中的空余时间锻炼身体。 | I can do physical exercise in my spare time on most days of the week even if I can watch TV or play video games instead. |
| 4 | 即使室外非常炎热或寒冷，我仍然可以在每周大部分天数中锻炼身体。 | I can do physical exercise on most days of the week even if it is very hot or cold outside. |
| 5 | 我可以要求我最好的朋友和我一起在每周大部分天数中一起锻炼身体。 | I can ask my best friends to do physical exercise with me on most days of the week. |
| 6 | 即使在家里我仍然可以锻炼身体。 | I can do physical exercise even at home. |
| 7 | 我可以锻炼身体是因为我知道如何锻炼。 | I can do physical exercise because I know how to exercise. |
| 8 | 不管我有多忙，我仍然可以在每周大部分天数中的空闲时间锻炼身体。 | I can do physical exercise in my spare time on most days of the week no matter how busy I am. |
