# Supplementary material for: A psychological network analysis of parental and peer support on children’s football participation: the bridging role of self-efficacy
Source: Front Psychol. 2026 Jun 17;17:1818293. doi: 10.3389/fpsyg.2026.1818293 (PMC13319038; doi:10.3389/fpsyg.2026.1818293)
Supplement: Supplementary file 2 [file Supplementary_file_2.DOCX]

## Part 1: main.R

# ============================================================================

# main.R

# ============================================================================

# 清理环境

rm(list = ls())

# ============================================================================

# 1. 设置基础路径（请根据你的实际情况修改！）

# ============================================================================

BASE_DIR <- "XXX"

DATA_PATH <- "XXX"

# 检查路径

if (!dir.exists(BASE_DIR)) stop("BASE_DIR 不存在：", BASE_DIR)

if (!file.exists(DATA_PATH)) stop("DATA_PATH 不存在：", DATA_PATH)

# ============================================================================

# 2. 创建输出目录（确保各模块结果存放路径存在）

# ============================================================================

OUTPUT_BASE <- file.path(BASE_DIR, "output")

dir.create(OUTPUT_BASE, showWarnings = FALSE, recursive = TRUE)

for (d in c("01_Results", "02_Results", "03_Results")) {

dir.create(file.path(OUTPUT_BASE, d), showWarnings = FALSE, recursive = TRUE)

}

cat("输出根目录：", OUTPUT_BASE, "\n\n")

# ============================================================================

# 3. 依次运行脚本（使用绝对路径）

# ============================================================================

cat("开始运行全部分析...\n\n")

source(file.path(BASE_DIR, "01_descriptive_correlation_reliability_validity.R"))

cat("✓ 01 脚本完成\n\n")

source(file.path(BASE_DIR, "02_psychological_network_analysis.R"))

cat("✓ 02 脚本完成\n\n")

source(file.path(BASE_DIR, "03_group_comparison.R"))

cat("✓ 03 脚本完成\n\n")

cat("所有分析运行完毕！结果保存在：\n")

cat(" - 01_Results: ", file.path(OUTPUT_BASE, "01_Results"), "\n")

cat(" - 02_Results: ", file.path(OUTPUT_BASE, "02_Results"), "\n")

cat(" - 03_Results: ", file.path(OUTPUT_BASE, "03_Results"), "\n")

## Part 2: 01_descriptive_correlation_reliability_validity.R

# ============================================================================

# 01_descriptive_correlation_reliability_validity.R

# ============================================================================

# ============================================================================

# 1. 环境配置与包加载

# ============================================================================

cat("\n", rep("=", 70), "\n", sep = "")

cat("PSYCHOMETRIC ANALYSIS\n")

cat(rep("=", 70), "\n\n", sep = "")

# 必要的包列表

required_packages <- c(

"tidyverse", "psych", "lavaan", "semTools", "semPlot",

"ggplot2", "corrplot", "MVN", "moments", "kableExtra",

"officer", "flextable", "openxlsx"

)

# 安装缺失的包

new_packages <- required_packages[!(required_packages %in% installed.packages()[,"Package"])]

if(length(new_packages) > 0) {

install.packages(new_packages)

}

# 加载包

suppressPackageStartupMessages({

library(tidyverse)

library(psych)

library(lavaan)

library(semTools)

library(semPlot)

library(ggplot2)

library(corrplot)

library(MVN)

library(moments)

library(kableExtra)

library(officer)

library(flextable)

library(openxlsx)

})

# ============================================================================

# 2. 路径设置

# ============================================================================

# 请根据你的实际情况修改这些路径

BASE_DIR <- "XXX"

DATA_PATH <- "XXX"

OUTPUT_DIR <- file.path(BASE_DIR, "output")

# 创建输出目录结构 - 只保留01_Results文件夹

dir.create(OUTPUT_DIR, showWarnings = FALSE, recursive = TRUE)

RESULTS_DIR <- file.path(OUTPUT_DIR, "01_Results")

dir.create(RESULTS_DIR, showWarnings = FALSE, recursive = TRUE)

# 在RESULTS_DIR下创建子文件夹以保持组织

sub_dirs <- c("01_descriptives", "02_reliability", "03_cfa",

"04_validity", "05_plots", "06_reports")

for(sub_dir in sub_dirs) {

dir.create(file.path(RESULTS_DIR, sub_dir), showWarnings = FALSE)

}

cat(sprintf("\n所有结果将保存至: %s\n", RESULTS_DIR))

# ============================================================================

# 3. 变量定义与构念映射

# ============================================================================

variable_names <- c(

"Grade", "Gender", "Age",

"G1", "G2", "G3", "G4",

"F1", "F2", "F3", "F4", "F5", "F6", "F7", "F8", "F9", "F10", "F11", "F12",

"S1", "S2", "S3", "S4", "S5", "S6", "S7", "S8",

"PA1", "PA2", "PA", "PS", "OS", "FS", "SE", "ZPA"

)

# 构念定义 - 统一使用无空格的变量名（用下划线连接）

construct_mapping <- list(

Parental_Support = list(

chinese = "父母支持",

english = "Parental_Support", # 使用下划线，避免空格问题

items = c("F1", "F2", "F3", "F4"),

type = "likert",

description = "父母情感性支持"

),

Peer_Support = list(

chinese = "同伴支持",

english = "Peer_Support",

items = c("F9", "F10", "F11", "F12"),

type = "likert",

description = "同伴工具性与情感性支持"

),

Self_Efficacy = list(

chinese = "自我效能感",

english = "Self_Efficacy",

items = paste0("S", 1:8),

type = "likert",

description = "运动自我效能感"

),

Football_Participation = list(

chinese = "足球参与",

english = "Football_Participation",

items = c("PA1", "PA2"),

type = "behavioral",

description = "每周参与天数与时长"

)

)

# ============================================================================

# 4. 数据加载与预处理函数

# ============================================================================

load_and_preprocess_data <- function(data_path, var_names) {

cat("\n[Step 1] Loading and preprocessing data...\n")

# 读取数据（无表头）

df <- read.csv(data_path, header = FALSE)

# 列数检查

if(ncol(df) != length(var_names)) {

stop(sprintf("列数不匹配！数据有 %d 列，变量定义有 %d 列", ncol(df), length(var_names)))

}

# 设置列名

colnames(df) <- var_names

# 将999转换为NA

df[df == 999] <- NA

# 基本信息

cat(sprintf("原始样本量: N = %d\n", nrow(df)))

cat(sprintf("变量数量: K = %d\n", ncol(df)))

# 处理性别因子

df <- df %>%

mutate(

Gender = factor(Gender,

levels = c(1, 2),

labels = c("Male", "Female")),

# 确保数值型变量

PA1 = as.numeric(PA1),

PA2 = as.numeric(PA2)

)

# 创建构念得分

cat("\nCreating construct scores...\n")

for(construct in names(construct_mapping)) {

con_def <- construct_mapping[[construct]]

items <- con_def$items

english_name <- con_def$english

# 检查所有题目是否存在于数据中

available_items <- items[items %in% names(df)]

if(length(available_items) > 0) {

if(con_def$type == "likert") {

# 李克特量表：计算均值

df[[english_name]] <- rowMeans(df[, available_items, drop = FALSE], na.rm = TRUE)

cat(sprintf(" Created %s (%s) from %d items\n", english_name, con_def$type, length(available_items)))

} else {

# 行为变量：创建组合指标（天数 × 时长）

if(all(c("PA1", "PA2") %in% available_items)) {

df[[english_name]] <- df$PA1 * df$PA2

cat(sprintf(" Created %s (%s) from PA1 * PA2\n", english_name, con_def$type))

} else {

# 如果只有单个指标，使用可用指标

df[[english_name]] <- df[[available_items[1]]]

cat(sprintf(" Created %s (%s) from single item %s\n", english_name, con_def$type, available_items[1]))

}

}

} else {

cat(sprintf(" Warning: No items available for %s\n", english_name))

}

}

# 对足球参与变量进行标准化（用于CFA）

if("PA1" %in% names(df) && "PA2" %in% names(df)) {

df <- df %>%

mutate(

PA1_std = as.numeric(scale(PA1)),

PA2_std = as.numeric(scale(PA2))

)

cat(" Created standardized versions: PA1_std, PA2_std\n")

}

# 显示创建的构念变量

construct_vars <- sapply(construct_mapping, function(x) x$english)

existing_constructs <- construct_vars[construct_vars %in% names(df)]

cat("\n成功创建的构念变量:\n")

for(con in existing_constructs) {

non_na <- sum(!is.na(df[[con]]))

cat(sprintf(" %s: n = %d (%.1f%% complete)\n",

con, non_na, non_na/nrow(df)*100))

}

cat("\n✓ 数据预处理完成\n")

cat(sprintf("最终分析样本量: N = %d (包含缺失值)\n", nrow(df)))

return(df)

}

# ============================================================================

# 5. 描述性统计分析函数

# ============================================================================

analyze_descriptives <- function(data, results_dir) {

cat("\n[Step 2] Computing descriptive statistics...\n")

# 获取所有构念变量

construct_vars <- sapply(construct_mapping, function(x) x$english)

existing_constructs <- construct_vars[construct_vars %in% names(data)]

# 获取所有原始题目

all_items <- unlist(lapply(construct_mapping, function(x) x$items))

existing_items <- all_items[all_items %in% names(data)]

# 综合描述统计

desc_vars <- c(existing_items, existing_constructs)

if(length(desc_vars) == 0) {

stop("No variables available for descriptive statistics!")

}

desc_stats <- data %>%

select(all_of(desc_vars)) %>%

psych::describe() %>%

as.data.frame() %>%

rownames_to_column("Variable") %>%

select(Variable, n, mean, sd, median, min, max, skew, kurtosis, se)

# 添加变量类型信息

desc_stats <- desc_stats %>%

mutate(

Type = case_when(

Variable %in% existing_constructs ~ "Construct",

Variable %in% c("PA1", "PA2") ~ "Behavioral",

TRUE ~ "Item"

)

)

# 保存结果到01_Results/01_descriptives

write.csv(desc_stats,

file.path(results_dir, "01_descriptives/descriptive_statistics.csv"),

row.names = FALSE)

cat(sprintf("✓ 描述性统计完成 (%d 个变量)\n", nrow(desc_stats)))

return(desc_stats)

}

# ============================================================================

# 6. 相关矩阵分析函数（修复：星号放在右上角）

# ============================================================================

analyze_correlations <- function(data, results_dir) {

cat("\n[Step 3] Computing correlation matrix...\n")

# 获取构念变量（排除人口学变量Grade, Gender, Age）

construct_vars <- sapply(construct_mapping, function(x) x$english)

existing_constructs <- construct_vars[construct_vars %in% names(data)]

# 确保排除人口学变量

demographic_vars <- c("Grade", "Gender", "Age")

existing_constructs <- existing_constructs[!existing_constructs %in% demographic_vars]

cat("用于相关分析的构念:\n")

print(existing_constructs)

if(length(existing_constructs) < 2) {

stop(sprintf("需要至少2个构念进行相关分析，当前只有 %d 个", length(existing_constructs)))

}

# 选择数据

cor_data <- data %>%

select(all_of(existing_constructs))

# 检查数据

cat(sprintf("相关分析数据框维度: %d 行, %d 列\n", nrow(cor_data), ncol(cor_data)))

cat("各变量非缺失值数量:\n")

print(colSums(!is.na(cor_data)))

# 计算相关矩阵

cor_result <- corr.test(cor_data, use = "pairwise", method = "pearson", adjust = "none")

R <- cor_result$r

P <- cor_result$p

# 保存相关矩阵到01_Results/01_descriptives

write.csv(R, file.path(results_dir, "01_descriptives/correlation_matrix.csv"))

write.csv(P, file.path(results_dir, "01_descriptives/correlation_pvalues.csv"))

# 创建相关热图

if(ncol(R) > 1) {

# 主热图：同时显示颜色和数字，星号在右上角

png(file.path(results_dir, "05_plots/correlation_heatmap.png"),

width = 3000, height = 2800, res = 300)

# 绘制热图

corrplot(R, method = "color", type = "upper",

tl.col = "black",

tl.srt = 45,

tl.cex = 1.2,

addCoef.col = "black",

number.cex = 1.2,

col = colorRampPalette(c("#6D9EC1", "white", "#E46726"))(200),

addgrid.col = "grey90",

title = "Correlation Matrix of Research Constructs",

mar = c(0,0,2,0),

cl.cex = 1.0)

# 添加显著性标记（星号）- 放在数字的右上角

n <- ncol(R)

for (i in 1:n) {

for (j in i:n) {

if (i != j && !is.na(P[i,j])) {

stars <- ifelse(P[i,j] < 0.001, "***",

ifelse(P[i,j] < 0.01, "**",

ifelse(P[i,j] < 0.05, "*", "")))

if(stars != "") {

# 修正：将星号放在数字的右上角

# 使用pos = 3（上方）并调整水平和垂直偏移

x_pos <- j + 0.15 # 向右偏移

y_pos <- n - i + 1 + 0.1 # 向上偏移

text(x_pos, y_pos, stars,

cex = 0.9, # 星号大小

col = "red",

adj = c(0, 0)) # 左下角对齐

}

}

}

}

dev.off()

cat("✓ 相关热图已保存（星号在数字右上角）\n")

# 创建一个带相关系数的表格用于报告

cor_table <- R

for(i in 1:nrow(R)) {

for(j in 1:ncol(R)) {

if(i != j && !is.na(P[i,j])) {

stars <- ifelse(P[i,j] < 0.001, "***",

ifelse(P[i,j] < 0.01, "**",

ifelse(P[i,j] < 0.05, "*", "")))

cor_table[i,j] <- paste0(round(R[i,j], 3), stars)

} else {

cor_table[i,j] <- round(R[i,j], 3)

}

}

}

write.csv(cor_table,

file.path(results_dir, "01_descriptives/correlation_matrix_with_significance.csv"))

} else {

cat("警告：因子数量不足，跳过相关热图\n")

}

cat("✓ 相关分析完成\n")

return(list(R = R, P = P))

}

# ============================================================================

# 7. 信度分析函数

# ============================================================================

analyze_reliability <- function(data, results_dir) {

cat("\n[Step 4] Testing reliability (Alpha & Omega)...\n")

reliability_results <- list()

for(construct in names(construct_mapping)) {

con_def <- construct_mapping[[construct]]

# 只分析李克特量表（多题目）

if(con_def$type == "likert" && length(con_def$items) > 1) {

items <- con_def$items

available_items <- items[items %in% names(data)]

if(length(available_items) > 1) {

item_data <- data[, available_items, drop = FALSE]

item_data <- item_data[complete.cases(item_data), ]

if(nrow(item_data) > 5) {

# Cronbach's Alpha

alpha_result <- psych::alpha(item_data, check.keys = TRUE)

alpha_raw <- alpha_result$total$raw_alpha

alpha_std <- alpha_result$total$std.alpha

# 计算平均相关系数

cor_mat <- cor(item_data, use = "pairwise")

mean_r <- mean(cor_mat[lower.tri(cor_mat)], na.rm = TRUE)

# 基于Spearman-Brown的Omega估计

if(length(available_items) >= 3) {

omega_est <- (length(available_items) * mean_r) / (1 + (length(available_items) - 1) * mean_r)

} else {

omega_est <- mean_r

}

reliability_results[[construct]] <- data.frame(

Construct = con_def$english,

Chinese = con_def$chinese,

Items = paste(available_items, collapse = ", "),

N_Items = length(available_items),

N_Valid = nrow(item_data),

Cronbach_Alpha = round(alpha_raw, 3),

Std_Alpha = round(alpha_std, 3),

Mean_InterItem_r = round(mean_r, 3),

Omega_est = round(omega_est, 3),

stringsAsFactors = FALSE

)

cat(sprintf(" %s: Alpha = %.3f, Omega_est = %.3f (n = %d)\n",

con_def$english, alpha_raw, omega_est, nrow(item_data)))

}

}

}

}

# 合并结果

if(length(reliability_results) > 0) {

rel_df <- bind_rows(reliability_results)

# 保存到01_Results/02_reliability

write.csv(rel_df,

file.path(results_dir, "02_reliability/reliability_analysis.csv"),

row.names = FALSE)

cat("✓ 信度分析完成\n")

return(rel_df)

} else {

cat("警告：没有足够的变量进行信度分析\n")

return(data.frame())

}

}

# ============================================================================

# 8. CFA分析函数

# ============================================================================

run_cfa_analysis <- function(data, results_dir) {

cat("\n[Step 5] Running Confirmatory Factor Analysis with FIML...\n")

# 检查必要变量是否存在

required_vars <- c("F1", "F2", "F3", "F4", "F9", "F10", "F11", "F12",

"S1", "S2", "S3", "S4", "S5", "S6", "S7", "S8",

"PA1_std", "PA2_std")

missing_vars <- required_vars[!required_vars %in% names(data)]

if(length(missing_vars) > 0) {

cat("警告：以下变量不存在于数据中:\n")

print(missing_vars)

cat("将使用可用变量拟合简化模型\n")

# 构建可用变量的模型

available_parental <- intersect(c("F1", "F2", "F3", "F4"), names(data))

available_peer <- intersect(c("F9", "F10", "F11", "F12"), names(data))

available_self <- intersect(paste0("S", 1:8), names(data))

available_football <- intersect(c("PA1_std", "PA2_std"), names(data))

cfa_model <- paste0(

'Parental_Support =~ ', paste(available_parental, collapse = " + "), '\n',

'Peer_Support =~ ', paste(available_peer, collapse = " + "), '\n',

'Self_Efficacy =~ ', paste(available_self, collapse = " + "), '\n',

'Football_Participation =~ ', paste(available_football, collapse = " + ")

)

} else {

# 完整模型

cfa_model <- '

Parental_Support =~ F1 + F2 + F3 + F4

Peer_Support =~ F9 + F10 + F11 + F12

Self_Efficacy =~ S1 + S2 + S3 + S4 + S5 + S6 + S7 + S8

Football_Participation =~ PA1_std + PA2_std

'

}

cat("CFA模型:\n")

cat(cfa_model, "\n")

# 拟合CFA模型

fit <- cfa(cfa_model,

data = data,

estimator = "MLR",

missing = "fiml",

std.lv = TRUE)

# 模型拟合指数

fit_indices <- c("chisq", "df", "pvalue",

"cfi", "tli",

"rmsea", "rmsea.ci.lower", "rmsea.ci.upper", "rmsea.pvalue",

"srmr",

"aic", "bic")

fit_measures <- fitMeasures(fit, fit_indices)

# 创建拟合指数数据框

fit_df <- data.frame(

Index = names(fit_measures),

Value = round(as.numeric(fit_measures), 3)

)

# 保存拟合指数到01_Results/03_cfa

write.csv(fit_df,

file.path(results_dir, "03_cfa/cfa_fit_indices.csv"),

row.names = FALSE)

# 提取标准化因子载荷

std_loadings <- tryCatch({

standardizedSolution(fit) %>%

filter(op == "=~") %>%

select(Latent = lhs, Indicator = rhs,

Estimate = est.std, SE = se,

Z = z, Pvalue = pvalue,

CI_lower = ci.lower, CI_upper = ci.upper) %>%

mutate(across(where(is.numeric), ~round(., 3)))

}, error = function(e) {

cat("提取标准化载荷时出错:", e$message, "\n")

return(data.frame())

})

if(nrow(std_loadings) > 0) {

write.csv(std_loadings,

file.path(results_dir, "03_cfa/standardized_loadings.csv"),

row.names = FALSE)

}

# 计算AVE和CR

ave_cr_df <- tryCatch({

aves <- semTools::AVE(fit)

rel_sem <- semTools::compRelSEM(fit)

data.frame(

Construct = names(aves),

AVE = round(as.numeric(aves), 3),

CR = round(as.numeric(rel_sem), 3)

)

}, error = function(e) {

cat("计算AVE/CR时出错:", e$message, "\n")

return(data.frame())

})

if(nrow(ave_cr_df) > 0) {

write.csv(ave_cr_df,

file.path(results_dir, "04_validity/ave_cr_results.csv"),

row.names = FALSE)

}

cat("✓ CFA分析完成\n")

return(list(

fit = fit,

fit_measures = fit_measures,

loadings = std_loadings,

ave_cr = ave_cr_df

))

}

# ============================================================================

# 9. 区分效度检验函数

# ============================================================================

test_discriminant_validity <- function(fit, results_dir) {

cat("\n[Step 6] Testing discriminant validity...\n")

# 获取因子相关矩阵

cor_mat <- tryCatch({

lavInspect(fit, "cor.lv")

}, error = function(e) {

cat("获取因子相关矩阵时出错:", e$message, "\n")

return(matrix())

})

if(length(cor_mat) > 1) {

# 获取AVE值

aves <- tryCatch({

semTools::AVE(fit)

}, error = function(e) {

return(rep(NA, ncol(cor_mat)))

})

# Fornell-Larcker准则

fl_results <- data.frame(

Factor = rownames(cor_mat),

Sqrt_AVE = round(sqrt(aves), 3)

)

# 添加最大相关系数

for(i in 1:nrow(fl_results)) {

other_cors <- cor_mat[i, -i]

fl_results$Max_Correlation[i] <- round(max(abs(other_cors), na.rm = TRUE), 3)

fl_results$FL_Passed[i] <- fl_results$Sqrt_AVE[i] > fl_results$Max_Correlation[i]

}

# 保存结果到01_Results/04_validity

write.csv(fl_results,

file.path(results_dir, "04_validity/fornell_larcker_results.csv"),

row.names = FALSE)

# 保存因子相关矩阵

write.csv(cor_mat,

file.path(results_dir, "04_validity/latent_correlations.csv"))

cat("✓ 区分效度检验完成\n")

return(list(fl_results = fl_results, correlations = cor_mat))

} else {

cat("警告：无法计算区分效度\n")

return(list(fl_results = data.frame(), correlations = matrix()))

}

}

# ============================================================================

# 10. 共同方法偏差检验

# ============================================================================

test_common_method_bias <- function(data, results_dir) {

cat("\n[Step 7] Testing common method bias...\n")

# Harman单因子检验

all_items <- unlist(lapply(construct_mapping, function(x) {

if(x$type == "likert") return(x$items)

}))

available_items <- all_items[all_items %in% names(data)]

if(length(available_items) > 3) {

# 提取所有李克特题目

likert_data <- data[, available_items, drop = FALSE]

likert_data <- likert_data[complete.cases(likert_data), ]

if(nrow(likert_data) > 10) {

# 未旋转的EFA

efa_unrotated <- tryCatch({

psych::fa(likert_data, nfactors = 1, fm = "ml")

}, error = function(e) {

cat("EFA分析出错:", e$message, "\n")

return(NULL)

})

if(!is.null(efa_unrotated) && !is.null(efa_unrotated$Vaccounted)) {

cmb_variance <- efa_unrotated$Vaccounted[2, 1] * 100

cmb_result <- data.frame(

Test = "Harman's Single Factor Test",

First_Factor_Variance = round(cmb_variance, 2),

Interpretation = ifelse(cmb_variance < 50,

"No severe common method bias",

"Potential common method bias")

)

} else {

cmb_result <- data.frame(

Test = "Harman's Single Factor Test",

First_Factor_Variance = NA,

Interpretation = "EFA failed to converge"

)

}

} else {

cmb_result <- data.frame(

Test = "Harman's Single Factor Test",

First_Factor_Variance = NA,

Interpretation = "Insufficient complete cases"

)

}

} else {

cmb_result <- data.frame(

Test = "Harman's Single Factor Test",

First_Factor_Variance = NA,

Interpretation = "Insufficient items"

)

}

# 保存结果到01_Results/04_validity

write.csv(cmb_result,

file.path(results_dir, "04_validity/common_method_bias.csv"),

row.names = FALSE)

cat("✓ 共同方法偏差检验完成\n")

return(cmb_result)

}

# ============================================================================

# 11. 生成分析报告

# ============================================================================

generate_comprehensive_report <- function(desc_stats, rel_results,

cfa_results, validity_results,

cmb_results, results_dir) {

cat("\n[Step 8] Generating comprehensive report...\n")

# 创建Word文档

doc <- read_docx()

# 添加标题

doc <- doc %>%

body_add_par("心理测量学分析报告", style = "heading 1") %>%

body_add_par(paste("生成时间:", Sys.time()), style = "Normal") %>%

body_add_par("", style = "Normal")

# 1. 描述性统计

if(nrow(desc_stats) > 0) {

desc_table <- desc_stats %>%

select(Variable, Type, n, mean, sd, skew, kurtosis) %>%

mutate(across(where(is.numeric) & !matches("Variable|Type"), ~round(., 3))) %>%

flextable() %>%

theme_apa() %>%

autofit()

doc <- doc %>%

body_add_par("1. 描述性统计", style = "heading 2") %>%

body_add_flextable(desc_table) %>%

body_add_par("", style = "Normal")

}

# 2. 信度分析

if(nrow(rel_results) > 0) {

rel_table <- rel_results %>%

select(Construct, Chinese, N_Items, N_Valid, Cronbach_Alpha, Omega_est) %>%

mutate(across(where(is.numeric), ~round(., 3))) %>%

flextable() %>%

theme_apa() %>%

autofit()

doc <- doc %>%

body_add_par("2. 信度分析", style = "heading 2") %>%

body_add_flextable(rel_table) %>%

body_add_par("", style = "Normal")

}

# 3. CFA拟合指数

if(length(cfa_results$fit_measures) > 0) {

fit_table <- data.frame(

Fit_Index = c("χ²", "df", "p", "CFI", "TLI", "RMSEA", "SRMR"),

Value = round(c(

cfa_results$fit_measures["chisq"],

cfa_results$fit_measures["df"],

cfa_results$fit_measures["pvalue"],

cfa_results$fit_measures["cfi"],

cfa_results$fit_measures["tli"],

cfa_results$fit_measures["rmsea"],

cfa_results$fit_measures["srmr"]

), 3)

) %>%

flextable() %>%

theme_apa() %>%

autofit()

doc <- doc %>%

body_add_par("3. 验证性因素分析", style = "heading 2") %>%

body_add_par("3.1 模型拟合指数", style = "heading 3") %>%

body_add_flextable(fit_table) %>%

body_add_par("", style = "Normal")

}

# 4. 因子载荷

if(nrow(cfa_results$loadings) > 0) {

load_table <- cfa_results$loadings %>%

select(Latent, Indicator, Estimate, Pvalue) %>%

mutate(Pvalue = round(Pvalue, 3)) %>%

flextable() %>%

theme_apa() %>%

autofit()

doc <- doc %>%

body_add_par("3.2 标准化因子载荷", style = "heading 3") %>%

body_add_flextable(load_table) %>%

body_add_par("", style = "Normal")

}

# 5. AVE和CR

if(nrow(cfa_results$ave_cr) > 0) {

ave_cr_table <- cfa_results$ave_cr %>%

flextable() %>%

theme_apa() %>%

autofit()

doc <- doc %>%

body_add_par("4. 聚合效度 (AVE & CR)", style = "heading 2") %>%

body_add_flextable(ave_cr_table) %>%

body_add_par("", style = "Normal")

}

# 6. 区分效度

if(nrow(validity_results$fl_results) > 0) {

fl_table <- validity_results$fl_results %>%

flextable() %>%

theme_apa() %>%

autofit()

doc <- doc %>%

body_add_par("5. 区分效度 (Fornell-Larcker)", style = "heading 2") %>%

body_add_flextable(fl_table) %>%

body_add_par("", style = "Normal")

}

# 7. 共同方法偏差

if(nrow(cmb_results) > 0) {

cmb_table <- cmb_results %>%

flextable() %>%

theme_apa() %>%

autofit()

doc <- doc %>%

body_add_par("6. 共同方法偏差检验", style = "heading 2") %>%

body_add_flextable(cmb_table)

}

# 保存报告到01_Results/06_reports

tryCatch({

print(doc, target = file.path(results_dir, "06_reports/psychometric_report.docx"))

cat("✓ Word报告已保存\n")

}, error = function(e) {

cat("保存Word报告时出错:", e$message, "\n")

})

# 保存Excel格式数据到01_Results/06_reports

wb <- createWorkbook()

if(nrow(desc_stats) > 0) {

addWorksheet(wb, "Descriptives")

writeData(wb, "Descriptives", desc_stats)

}

if(nrow(rel_results) > 0) {

addWorksheet(wb, "Reliability")

writeData(wb, "Reliability", rel_results)

}

if(length(cfa_results$fit_measures) > 0) {

addWorksheet(wb, "Fit_Indices")

fit_df <- data.frame(

Index = names(cfa_results$fit_measures),

Value = as.numeric(cfa_results$fit_measures)

)

writeData(wb, "Fit_Indices", fit_df)

}

if(nrow(cfa_results$loadings) > 0) {

addWorksheet(wb, "Loadings")

writeData(wb, "Loadings", cfa_results$loadings)

}

if(nrow(cfa_results$ave_cr) > 0) {

addWorksheet(wb, "AVE_CR")

writeData(wb, "AVE_CR", cfa_results$ave_cr)

}

if(nrow(validity_results$fl_results) > 0) {

addWorksheet(wb, "Discriminant_Validity")

writeData(wb, "Discriminant_Validity", validity_results$fl_results)

}

if(nrow(cmb_results) > 0) {

addWorksheet(wb, "CMB")

writeData(wb, "CMB", cmb_results)

}

saveWorkbook(wb, file.path(results_dir, "06_reports/all_results.xlsx"), overwrite = TRUE)

cat("✓ Excel数据已保存\n")

}

# ============================================================================

# 12. 主分析流程

# ============================================================================

run_complete_analysis <- function() {

cat("\n", rep("=", 70), "\n", sep = "")

cat("STARTING COMPLETE PSYCHOMETRIC ANALYSIS (FIXED VERSION 2)\n")

cat(rep("=", 70), "\n\n", sep = "")

# 1. 加载数据

df <- load_and_preprocess_data(DATA_PATH, variable_names)

# 2. 描述性统计

desc_results <- analyze_descriptives(df, RESULTS_DIR)

# 3. 相关分析

cor_results <- analyze_correlations(df, RESULTS_DIR)

# 4. 信度分析

rel_results <- analyze_reliability(df, RESULTS_DIR)

# 5. CFA分析

cfa_results <- run_cfa_analysis(df, RESULTS_DIR)

# 6. 区分效度

validity_results <- test_discriminant_validity(cfa_results$fit, RESULTS_DIR)

# 7. 共同方法偏差

cmb_results <- test_common_method_bias(df, RESULTS_DIR)

# 8. 生成报告

generate_comprehensive_report(desc_results, rel_results,

cfa_results, validity_results,

cmb_results, RESULTS_DIR)

cat("\n", rep("=", 70), "\n", sep = "")

cat("ANALYSIS COMPLETED SUCCESSFULLY!\n")

cat(rep("=", 70), "\n\n", sep = "")

cat(sprintf("\n所有结果已保存至: %s\n", RESULTS_DIR))

cat("包含以下子文件夹：\n")

cat(" - 01_descriptives: 描述性统计和相关分析\n")

cat(" - 02_reliability: 信度分析\n")

cat(" - 03_cfa: 验证性因素分析\n")

cat(" - 04_validity: 效度分析\n")

cat(" - 05_plots: 图形文件\n")

cat(" - 06_reports: 综合报告\n")

return(list(

data = df,

descriptives = desc_results,

correlations = cor_results,

reliability = rel_results,

cfa = cfa_results,

validity = validity_results,

cmb = cmb_results

))

}

# ============================================================================

# 13. 执行分析

# ============================================================================

set.seed(2024)

results <- run_complete_analysis()

# 查看关键结果摘要

cat("\n关键结果摘要:\n")

cat(sprintf("样本量: N = %d (含缺失值)\n", nrow(results$data)))

if(!is.null(results$cfa$fit_measures)) {

cat(sprintf("CFI = %.3f, TLI = %.3f, RMSEA = %.3f\n",

results$cfa$fit_measures["cfi"],

results$cfa$fit_measures["tli"],

results$cfa$fit_measures["rmsea"]))

}

if(nrow(results$reliability) > 0) {

cat("\n信度系数:\n")

print(results$reliability %>% select(Construct, Cronbach_Alpha, Omega_est))

}

if(nrow(results$cfa$ave_cr) > 0) {

cat("\nAVE和CR:\n")

print(results$cfa$ave_cr)

}

## Part 3: 02_psychological_network_analysis.R

# ============================================================================

# 02_psychological_network_analysis.R

# ============================================================================

rm(list = ls())

cat("\n", rep("=", 70), "\n", sep = "")

cat("PSYCHOLOGICAL NETWORK ANALYSIS - FULL SOURCE CODE (ABSOLUTELY COMPLETE)\n")

cat(rep("=", 70), "\n\n", sep = "")

# ============================================================================

# 1. 环境配置与包加载

# ============================================================================

cat("[1] 正在加载所有必要的R包...\n")

suppressPackageStartupMessages({

library(qgraph) # 网络估计与绘图

library(bootnet) # 稳定性分析

library(networktools) # 聚类系数与桥梁分析

library(igraph) # 高级图论计算

library(ggplot2) # 数据可视化

library(dplyr) # 数据清洗

library(tidyr) # 数据整理

library(RColorBrewer) # 颜色配置

library(psych) # 统计计算

})

# 检查并安装moments包（用于描述性统计）

if (!requireNamespace("moments", quietly = TRUE)) {

cat(" ⚠️ 正在安装moments包...\n")

install.packages("moments")

}

library(moments)

# ============================================================================

# 2. 路径设置

# ============================================================================

cat("[2] 正在设置工作路径与创建文件夹...\n")

BASE_DIR <- "XXX"

DATA_PATH <- "XXX"

OUTPUT_DIR <- file.path(BASE_DIR, "output/02_Results")

# 创建所有需要的文件夹

dirs <- c(

"01_networks/construct_level", "01_networks/item_level",

"02_centrality/construct_level", "02_centrality/item_level",

"03_bridge_analysis", "04_edge_weights", "05_stability",

"06_clustering", "07_tables", "08_report"

)

for(d in file.path(OUTPUT_DIR, dirs)) {

dir.create(d, recursive = TRUE, showWarnings = FALSE)

}

# ============================================================================

# 3. 数据加载与预处理

# ============================================================================

cat("[3] 正在加载并预处理数据...\n")

variable_names <- c(

"Grade", "Gender", "Age",

"G1", "G2", "G3", "G4",

"F1", "F2", "F3", "F4", "F5", "F6", "F7", "F8", "F9", "F10", "F11", "F12",

"S1", "S2", "S3", "S4", "S5", "S6", "S7", "S8",

"PA1", "PA2", "PA", "PS", "OS", "FS", "SE", "ZPA"

)

construct_mapping <- list(

Parental_Support = list(

english = "Parental_Support",

display = "Parental Support",

items = c("F1", "F2", "F3", "F4"),

color = "#E69F00"

),

Peer_Support = list(

english = "Peer_Support",

display = "Peer Support",

items = c("F9", "F10", "F11", "F12"),

color = "#56B4E9"

),

Self_Efficacy = list(

english = "Self_Efficacy",

display = "Self-Efficacy",

items = paste0("S", 1:8),

color = "#009E73"

),

Football_Participation = list(

english = "Football_Participation",

display = "Football Participation",

items = c("PA1", "PA2"),

color = "#F0E442"

)

)

display_names <- setNames(

sapply(construct_mapping, function(x) x$display),

sapply(construct_mapping, function(x) x$english)

)

# 读取数据

raw_data <- read.csv(DATA_PATH, header = FALSE)

colnames(raw_data) <- variable_names[1:ncol(raw_data)]

raw_data[raw_data == 999] <- NA

# 提取条目数据并填补中位数

all_items_list <- unlist(lapply(construct_mapping, function(x) x$items))

item_data_clean <- raw_data[, all_items_list[all_items_list %in% names(raw_data)]]

item_data_complete <- as.data.frame(lapply(item_data_clean, function(x) {

x[is.na(x)] <- median(x, na.rm = TRUE)

return(x)

}))

n <- nrow(item_data_complete)

# ============================================================================

# 4. 构念层数据准备

# ============================================================================

cat("[4] 正在计算构念层均值...\n")

construct_data <- data.frame(row.names = 1:n)

for(con in names(construct_mapping)) {

items_to_average <- construct_mapping[[con]]$items

construct_data[[construct_mapping[[con]]$english]] <-

rowMeans(item_data_complete[, items_to_average], na.rm = TRUE)

}

# ============================================================================

# 4.5 描述性统计分析（Table 01）

# ============================================================================

cat("[4.5] 正在计算描述性统计...\n")

# 条目层描述性统计

item_desc <- data.frame(

Variable = colnames(item_data_complete),

N = n,

Mean = round(colMeans(item_data_complete, na.rm = TRUE), 2),

SD = round(apply(item_data_complete, 2, sd, na.rm = TRUE), 2),

Min = round(apply(item_data_complete, 2, min, na.rm = TRUE), 2),

Max = round(apply(item_data_complete, 2, max, na.rm = TRUE), 2),

Skew = round(apply(item_data_complete, 2, function(x) skewness(x, na.rm = TRUE)), 2),

Kurtosis = round(apply(item_data_complete, 2, function(x) kurtosis(x, na.rm = TRUE)), 2)

)

# 添加构念信息

item_map_stack <- stack(lapply(construct_mapping, function(x) x$items))

item_desc$Construct <- item_map_stack$ind[match(item_desc$Variable, item_map_stack$values)]

# 重新排列列

item_desc <- item_desc[, c("Variable", "Construct", "N", "Mean", "SD", "Min", "Max", "Skew", "Kurtosis")]

# 保存条目层描述性统计

write.csv(item_desc,

file.path(OUTPUT_DIR, "07_tables/01_item_descriptives.csv"),

row.names = FALSE)

# 构念层描述性统计

construct_desc <- data.frame(

Construct = names(construct_data),

N = n,

Mean = round(colMeans(construct_data, na.rm = TRUE), 2),

SD = round(apply(construct_data, 2, sd, na.rm = TRUE), 2),

Min = round(apply(construct_data, 2, min, na.rm = TRUE), 2),

Max = round(apply(construct_data, 2, max, na.rm = TRUE), 2),

Skew = round(apply(construct_data, 2, function(x) skewness(x, na.rm = TRUE)), 2),

Kurtosis = round(apply(construct_data, 2, function(x) kurtosis(x, na.rm = TRUE)), 2)

)

# 添加显示名称

construct_desc$Display <- display_names[construct_desc$Construct]

# 保存构念层描述性统计

write.csv(construct_desc,

file.path(OUTPUT_DIR, "07_tables/01_construct_descriptives.csv"),

row.names = FALSE)

cat(" ✓ 描述性统计表已保存至: 01_item_descriptives.csv 和 01_construct_descriptives.csv\n")

# ============================================================================

# 5. 网络估计

# ============================================================================

cat("[5] 正在估计网络结构 (EBICglasso)...\n")

estimate_network <- function(data) {

set.seed(2024)

cor_matrix <- cor_auto(data)

adj_matrix <- EBICglasso(

cor_matrix,

n = nrow(data),

gamma = 0.1,

penalize.diagonal = FALSE,

threshold = FALSE,

lambda.min.ratio = 0.001

)

rownames(adj_matrix) <- colnames(adj_matrix) <- colnames(data)

nz_edges <- sum(adj_matrix[upper.tri(adj_matrix)] != 0)

net_density <- nz_edges / (ncol(adj_matrix) * (ncol(adj_matrix) - 1) / 2)

e_stats <- list(

mean = mean(adj_matrix[adj_matrix != 0]),

positive = sum(adj_matrix > 0),

negative = sum(adj_matrix < 0)

)

return(list(

adj = adj_matrix,

density = net_density,

nonzero_edges = nz_edges,

edge_stats = e_stats

))

}

item_network <- estimate_network(item_data_complete)

adj_items <- item_network$adj

construct_network <- estimate_network(construct_data)

adj_constructs <- construct_network$adj

# ============================================================================

# 5.5 相关矩阵表（Table 02）

# ============================================================================

cat("[5.5] 正在保存相关矩阵...\n")

# 条目层相关矩阵

cor_items <- cor_auto(item_data_complete)

write.csv(round(cor_items, 3),

file.path(OUTPUT_DIR, "07_tables/02_item_correlations.csv"),

row.names = TRUE)

# 构念层相关矩阵

cor_constructs <- cor_auto(construct_data)

rownames(cor_constructs) <- colnames(cor_constructs) <- display_names[colnames(cor_constructs)]

write.csv(round(cor_constructs, 3),

file.path(OUTPUT_DIR, "07_tables/02_construct_correlations.csv"),

row.names = TRUE)

cat(" ✓ 相关矩阵表已保存至: 02_item_correlations.csv 和 02_construct_correlations.csv\n")

# ============================================================================

# 6. 中心性分析

# ============================================================================

cat("[6] 正在计算中心性指标...\n")

compute_centrality_df <- function(adj) {

cent_obj <- centrality(adj)

df <- data.frame(

Node = colnames(adj),

Strength = round(cent_obj$OutDegree, 3),

Closeness = round(cent_obj$Closeness, 3),

Betweenness = round(cent_obj$Betweenness, 3),

ExpectedInfluence = round(cent_obj$OutExpectedInfluence, 3)

)

return(df)

}

cent_items_df <- compute_centrality_df(adj_items)

cent_constructs_df <- compute_centrality_df(adj_constructs)

item_map_stack <- stack(lapply(construct_mapping, function(x) x$items))

cent_items_df$Construct <- item_map_stack$ind[match(cent_items_df$Node, item_map_stack$values)]

# ============================================================================

# 6.5 保存中心性指标表（Table 03）

# ============================================================================

cat("[6.5] 正在保存中心性指标表...\n")

# 条目层中心性表（已包含构念信息）

write.csv(cent_items_df,

file.path(OUTPUT_DIR, "07_tables/03_item_centrality.csv"),

row.names = FALSE)

# 构念层中心性表

cent_constructs_df_display <- cent_constructs_df

cent_constructs_df_display$Node <- display_names[cent_constructs_df_display$Node]

write.csv(cent_constructs_df_display,

file.path(OUTPUT_DIR, "07_tables/03_construct_centrality.csv"),

row.names = FALSE)

cat(" ✓ 中心性指标表已保存至: 03_item_centrality.csv 和 03_construct_centrality.csv\n")

# ============================================================================

# 7. 桥梁中心性分析

# ============================================================================

cat("[7] 正在进行桥梁分析...\n")

bridge_comm <- list()

for(i in 1:ncol(adj_constructs)) {

bridge_comm[[colnames(adj_constructs)[i]]] <- i

}

bridge_obj <- bridge(adj_constructs, communities = bridge_comm)

bridge_df <- data.frame(

Node = names(bridge_obj$`Bridge Strength`),

Bridge_Strength = round(as.numeric(bridge_obj$`Bridge Strength`), 3)

)

bridge_df$Display <- display_names[bridge_df$Node]

bridge_df <- bridge_df[order(-bridge_df$Bridge_Strength), ]

write.csv(bridge_df, file.path(OUTPUT_DIR, "07_tables/04_bridge_centrality.csv"), row.names = FALSE)

# ============================================================================

# 8. 最强连接识别

# ============================================================================

cat("[8] 正在提取最强连接...\n")

extract_top_edges <- function(adj, n_top = 20) {

edge_list <- data.frame()

for(i in 1:(nrow(adj)-1)) {

for(j in (i+1):nrow(adj)) {

if(adj[i,j] != 0) {

edge_list <- rbind(edge_list, data.frame(

from = rownames(adj)[i],

to = colnames(adj)[j],

weight = round(adj[i,j], 3)

))

}

}

}

return(edge_list[order(-abs(edge_list$weight)), ][1:min(n_top, nrow(edge_list)), ])

}

top_items <- extract_top_edges(adj_items)

top_constructs <- extract_top_edges(adj_constructs)

write.csv(top_items, file.path(OUTPUT_DIR, "07_tables/06_top_edges_item.csv"), row.names = FALSE)

write.csv(top_constructs, file.path(OUTPUT_DIR, "07_tables/05_top_edges_construct.csv"), row.names = FALSE)

# ============================================================================

# 9. 聚类系数分析 (WS, Barrat, Onnela, Zhang) - 同时输出图和表

# ============================================================================

cat("[9] 正在分析聚类系数 (四指标)...\n")

# 检查clusteringPlot函数是否可用

if (!exists("clusteringPlot")) {

stop("clusteringPlot 函数不存在，请检查 qgraph 包是否正确加载。")

}

# 强制对称化矩阵（避免浮点误差）

make_symmetric <- function(mat) {

(mat + t(mat)) / 2

}

adj_items_sym <- make_symmetric(adj_items)

adj_constructs_sym <- make_symmetric(adj_constructs)

# ============================================================================

# 自定义函数：计算四种聚类系数

# ============================================================================

compute_clustering_metrics <- function(adj, level_name) {

# 将邻接矩阵转换为igraph对象

adj_abs <- (abs(adj) + t(abs(adj))) / 2 # 确保对称

g <- graph_from_adjacency_matrix(adj_abs, mode = "undirected", weighted = TRUE, diag = FALSE)

# 1. WS指数 (Watts-Strogatz) - 基于二值网络

g_binary <- graph_from_adjacency_matrix(adj_abs > 0, mode = "undirected", diag = FALSE)

ws_values <- transitivity(g_binary, type = "local")

ws_global <- transitivity(g_binary, type = "global")

# 2. Barrat指数 - 加权网络的聚类系数

barrat_values <- transitivity(g, type = "barrat")

barrat_global <- mean(barrat_values, na.rm = TRUE)

# 3. Onnela指数 - 另一种加权聚类系数

onnela_values <- vector("numeric", length = vcount(g))

names(onnela_values) <- V(g)$name

for(i in 1:vcount(g)) {

neighbors <- neighbors(g, i)

if(length(neighbors) >= 2) {

tri_weights <- c()

for(j in 1:(length(neighbors)-1)) {

for(k in (j+1):length(neighbors)) {

if(adj_abs[neighbors[j], neighbors[k]] > 0) {

w_ij <- adj_abs[i, neighbors[j]]

w_ik <- adj_abs[i, neighbors[k]]

w_jk <- adj_abs[neighbors[j], neighbors[k]]

tri_weights <- c(tri_weights, (w_ij * w_ik * w_jk)^(1/3))

}

}

}

if(length(tri_weights) > 0) {

onnela_values[i] <- mean(tri_weights, na.rm = TRUE)

} else {

onnela_values[i] <- 0

}

} else {

onnela_values[i] <- 0

}

}

onnela_global <- mean(onnela_values, na.rm = TRUE)

# 4. Zhang指数

zhang_values <- vector("numeric", length = vcount(g))

names(zhang_values) <- V(g)$name

for(i in 1:vcount(g)) {

neighbors <- neighbors(g, i)

if(length(neighbors) >= 2) {

tri_weights <- c()

for(j in 1:(length(neighbors)-1)) {

for(k in (j+1):length(neighbors)) {

w_ij <- adj_abs[i, neighbors[j]]

w_ik <- adj_abs[i, neighbors[k]]

w_jk <- adj_abs[neighbors[j], neighbors[k]]

tri_weights <- c(tri_weights, (w_ij * w_ik * w_jk)^(1/3) / (max(w_ij, w_ik, w_jk)))

}

}

if(length(tri_weights) > 0) {

zhang_values[i] <- mean(tri_weights, na.rm = TRUE)

} else {

zhang_values[i] <- 0

}

} else {

zhang_values[i] <- 0

}

}

zhang_global <- mean(zhang_values, na.rm = TRUE)

node_df <- data.frame(

Node = colnames(adj),

WS = round(ws_values, 3),

Barrat = round(barrat_values, 3),

Onnela = round(onnela_values, 3),

Zhang = round(zhang_values, 3)

)

global_df <- data.frame(

Level = level_name,

WS_Global = round(ws_global, 3),

Barrat_Global = round(barrat_global, 3),

Onnela_Global = round(onnela_global, 3),

Zhang_Global = round(zhang_global, 3)

)

return(list(

node_level = node_df,

global_level = global_df,

ws_values = ws_values,

barrat_values = barrat_values,

onnela_values = onnela_values,

zhang_values = zhang_values

))

}

# ============================================================================

# 绘图函数：使用clusteringPlot生成点线图

# ============================================================================

plot_clustering_figure <- function(adj, level_name, metrics_list) {

tryCatch({

png_path <- file.path(OUTPUT_DIR, paste0("06_clustering/01_", level_name, "_clustering_plot.png"))

png(png_path, width = 3000, height = 2000, res = 300)

clusteringPlot(adj,

include = c("WS", "Barrat", "Onnela", "Zhang"),

scale = "z-scores",

labels = colnames(adj),

theme_bw = TRUE)

dev.off()

cat("✓ 聚类系数点线图已保存至：", png_path, "\n")

}, error = function(e) {

cat("⚠️ clusteringPlot 失败：", e$message, "\n")

})

}

# ============================================================================

# 主程序：计算并保存条目层聚类系数

# ============================================================================

cat("\n--- 计算条目层聚类系数 ---\n")

item_metrics <- compute_clustering_metrics(adj_items_sym, "item")

write.csv(item_metrics$node_level, file.path(OUTPUT_DIR, "06_clustering/item_clustering_node_level.csv"), row.names = FALSE)

write.csv(item_metrics$global_level, file.path(OUTPUT_DIR, "06_clustering/item_clustering_global.csv"), row.names = FALSE)

item_clustering <- plot_clustering_figure(adj_items_sym, "item", item_metrics)

# ============================================================================

# 主程序：计算并保存构念层聚类系数

# ============================================================================

cat("\n--- 计算构念层聚类系数 ---\n")

construct_metrics <- compute_clustering_metrics(adj_constructs_sym, "construct")

write.csv(construct_metrics$node_level, file.path(OUTPUT_DIR, "06_clustering/construct_clustering_node_level.csv"), row.names = FALSE)

write.csv(construct_metrics$global_level, file.path(OUTPUT_DIR, "06_clustering/construct_clustering_global.csv"), row.names = FALSE)

construct_clustering <- plot_clustering_figure(adj_constructs_sym, "construct", construct_metrics)

# ============================================================================

# 生成汇总表格（四指标对比表）

# ============================================================================

cat("\n--- 生成四指标汇总表 ---\n")

item_summary <- data.frame(

Level = "Item",

Metric = c("WS", "Barrat", "Onnela", "Zhang"),

Global_Coefficient = round(c(

item_metrics$global_level$WS_Global,

item_metrics$global_level$Barrat_Global,

item_metrics$global_level$Onnela_Global,

item_metrics$global_level$Zhang_Global

), 3),

Mean_Local = round(c(

mean(item_metrics$ws_values, na.rm = TRUE),

mean(item_metrics$barrat_values, na.rm = TRUE),

mean(item_metrics$onnela_values, na.rm = TRUE),

mean(item_metrics$zhang_values, na.rm = TRUE)

), 3),

SD_Local = round(c(

sd(item_metrics$ws_values, na.rm = TRUE),

sd(item_metrics$barrat_values, na.rm = TRUE),

sd(item_metrics$onnela_values, na.rm = TRUE),

sd(item_metrics$zhang_values, na.rm = TRUE)

), 3)

)

construct_summary <- data.frame(

Level = "Construct",

Metric = c("WS", "Barrat", "Onnela", "Zhang"),

Global_Coefficient = round(c(

construct_metrics$global_level$WS_Global,

construct_metrics$global_level$Barrat_Global,

construct_metrics$global_level$Onnela_Global,

construct_metrics$global_level$Zhang_Global

), 3),

Mean_Local = round(c(

mean(construct_metrics$ws_values, na.rm = TRUE),

mean(construct_metrics$barrat_values, na.rm = TRUE),

mean(construct_metrics$onnela_values, na.rm = TRUE),

mean(construct_metrics$zhang_values, na.rm = TRUE)

), 3),

SD_Local = round(c(

sd(construct_metrics$ws_values, na.rm = TRUE),

sd(construct_metrics$barrat_values, na.rm = TRUE),

sd(construct_metrics$onnela_values, na.rm = TRUE),

sd(construct_metrics$zhang_values, na.rm = TRUE)

), 3)

)

clustering_summary <- rbind(item_summary, construct_summary)

cat("\n========== 聚类系数四指标汇总表 ==========\n")

print(clustering_summary)

cat("==========================================\n\n")

write.csv(clustering_summary, file.path(OUTPUT_DIR, "06_clustering/clustering_four_metrics_summary.csv"), row.names = FALSE)

# ============================================================================

# 更新item_clustering和construct_clustering对象

# ============================================================================

item_clustering <- list(

global_df = item_metrics$global_level,

global = list(

WS = item_metrics$global_level$WS_Global,

Barrat = item_metrics$global_level$Barrat_Global,

Onnela = item_metrics$global_level$Onnela_Global,

Zhang = item_metrics$global_level$Zhang_Global

),

node = item_metrics$node_level,

mean_local = mean(item_metrics$ws_values, na.rm = TRUE),

mean_local_all = list(

WS = mean(item_metrics$ws_values, na.rm = TRUE),

Barrat = mean(item_metrics$barrat_values, na.rm = TRUE),

Onnela = mean(item_metrics$onnela_values, na.rm = TRUE),

Zhang = mean(item_metrics$zhang_values, na.rm = TRUE)

)

)

construct_clustering <- list(

global_df = construct_metrics$global_level,

global = list(

WS = construct_metrics$global_level$WS_Global,

Barrat = construct_metrics$global_level$Barrat_Global,

Onnela = construct_metrics$global_level$Onnela_Global,

Zhang = construct_metrics$global_level$Zhang_Global

),

node = construct_metrics$node_level,

mean_local = mean(construct_metrics$ws_values, na.rm = TRUE),

mean_local_all = list(

WS = mean(construct_metrics$ws_values, na.rm = TRUE),

Barrat = mean(construct_metrics$barrat_values, na.rm = TRUE),

Onnela = mean(construct_metrics$onnela_values, na.rm = TRUE),

Zhang = mean(construct_metrics$zhang_values, na.rm = TRUE)

)

)

cat("\n✅ 聚类系数分析完成！\n")

# ============================================================================

# 10. 网络可视化 (条目层与构念层)

# ============================================================================

cat("[10] 正在生成网络图像...\n")

item_groups_plot <- list()

for(con in names(construct_mapping)) {

item_groups_plot[[construct_mapping[[con]]$display]] <- which(colnames(adj_items) %in% construct_mapping[[con]]$items)

}

png(file.path(OUTPUT_DIR, "01_networks/item_level/01_item_network_fixed.png"),

width = 3500, height = 2500, res = 300)

qgraph(adj_items,

layout = "spring",

groups = item_groups_plot,

color = sapply(construct_mapping, function(x) x$color),

vsize = 6,

label.cex = 1.5,

label.scale = FALSE,

edge.width = 0.8,

minimum = 0.005,

cut = 0.05,

legend = TRUE,

legend.cex = 1.0,

negDashed = TRUE,

title = "Item-Level Network")

dev.off()

png(file.path(OUTPUT_DIR, "01_networks/construct_level/01_construct_network.png"),

width = 3000, height = 2500, res = 300)

qgraph(adj_constructs,

layout = "circle",

color = sapply(construct_mapping, function(x) x$color),

vsize = 14,

label.cex = 1.8,

label.scale = FALSE,

labels = display_names,

edge.labels = FALSE,

title = "Construct-Level Network")

dev.off()

# 10.3 组合图：构念层与条目层并排显示（优化比例，条目层更大）

png(file.path(OUTPUT_DIR, "01_networks/combined_network.png"),

width = 8000, height = 3000, res = 300)

# 设置左右比例：左边18%，右边82%（可根据预览效果微调）

layout(matrix(c(1, 2), nrow = 1), widths = c(0.2, 0.2))

# 左图：构念层

par(mar = c(2, 2, 4, 2))

qgraph(adj_constructs,

layout = "circle",

color = sapply(construct_mapping, function(x) x$color),

vsize = 6,

label.cex = 1.4,

label.scale = FALSE,

edge.width = 0.8,

labels = display_names,

edge.labels = FALSE,

mar = c(5, 5, 5, 5))

# 右图：条目层（微调内部边距，让网络更靠左，为图例腾空间）

par(mar = c(2, 2, 4, 2))

qgraph(adj_items,

layout = "spring",

groups = item_groups_plot,

color = sapply(construct_mapping, function(x) x$color),

vsize = 6,

label.cex = 1.2,

label.scale = FALSE,

edge.width = 0.8,

minimum = 0.005,

cut = 0.05,

legend = TRUE,

legend.cex = 0.8,

negDashed = TRUE,

mar = c(5, 5, 5, 6)) # 左边距从5减到3，网络区域更宽；右边距8保留图例空间

dev.off()

# ============================================================================

# 11. 中心性绘图

# ============================================================================

cat("[11] 正在生成中心性图...\n")

png(file.path(OUTPUT_DIR, "02_centrality/item_level/01_item_centrality.png"), width = 3200, height = 2800, res = 300)

centralityPlot(adj_items, include = c("Strength", "Closeness", "Betweenness", "ExpectedInfluence"),

scale = "z-scores", theme_bw = TRUE)

dev.off()

png(file.path(OUTPUT_DIR, "02_centrality/construct_level/01_construct_centrality.png"), width = 3000, height = 2500, res = 300)

centralityPlot(adj_constructs, labels = display_names,

include = c("Strength", "Closeness", "Betweenness", "ExpectedInfluence"),

scale = "z-scores", theme_bw = TRUE)

dev.off()

# ============================================================================

# 11.5 社区发现分析

# ============================================================================

cat("[11.5] 正在进行社区发现分析...\n")

library(igraph)

adj_items_abs <- abs(adj_items)

adj_items_abs <- (adj_items_abs + t(adj_items_abs)) / 2

adj_constructs_abs <- abs(adj_constructs)

adj_constructs_abs <- (adj_constructs_abs + t(adj_constructs_abs)) / 2

g_items <- graph_from_adjacency_matrix(adj_items_abs, mode = "undirected", weighted = TRUE, diag = FALSE)

g_constructs <- graph_from_adjacency_matrix(adj_constructs_abs, mode = "undirected", weighted = TRUE, diag = FALSE)

# 三种社区发现算法

cat(" - 应用walktrap社区发现算法...\n")

set.seed(2024)

walktrap_items <- cluster_walktrap(g_items, steps = 4)

walktrap_constructs <- cluster_walktrap(g_constructs, steps = 4)

cat(" - 应用Louvain社区发现算法...\n")

set.seed(2024)

louvain_items <- cluster_louvain(g_items)

louvain_constructs <- cluster_louvain(g_constructs)

cat(" - 应用fast-greedy社区发现算法...\n")

set.seed(2024)

fgreedy_items <- cluster_fast_greedy(g_items)

fgreedy_constructs <- cluster_fast_greedy(g_constructs)

# 计算模块度

modularity_items <- data.frame(

Level = "Item",

Algorithm = c("Walktrap", "Louvain", "Fast-Greedy"),

Modularity = round(c(

modularity(walktrap_items),

modularity(louvain_items),

modularity(fgreedy_items)

), 3),

N_Communities = c(

length(unique(membership(walktrap_items))),

length(unique(membership(louvain_items))),

length(unique(membership(fgreedy_items)))

)

)

modularity_constructs <- data.frame(

Level = "Construct",

Algorithm = c("Walktrap", "Louvain", "Fast-Greedy"),

Modularity = round(c(

modularity(walktrap_constructs),

modularity(louvain_constructs),

modularity(fgreedy_constructs)

), 3),

N_Communities = c(

length(unique(membership(walktrap_constructs))),

length(unique(membership(louvain_constructs))),

length(unique(membership(fgreedy_constructs)))

)

)

modularity_summary <- rbind(modularity_items, modularity_constructs)

cat("\n========== 社区发现模块度 ==========\n")

print(modularity_summary)

cat("====================================\n\n")

write.csv(modularity_summary, file.path(OUTPUT_DIR, "07_tables/08_modularity_summary.csv"), row.names = FALSE)

# 创建成员关系表格

item_communities <- data.frame(

Node = V(g_items)$name,

Community_Louvain = membership(louvain_items),

Community_Walktrap = membership(walktrap_items),

Community_FastGreedy = membership(fgreedy_items)

)

item_map <- data.frame(

Node = unlist(lapply(construct_mapping, function(x) x$items)),

Construct = rep(names(construct_mapping), times = sapply(construct_mapping, function(x) length(x$items)))

)

item_communities <- merge(item_communities, item_map, by = "Node", all.x = TRUE)

item_communities <- item_communities[, c("Node", "Construct", "Community_Louvain", "Community_Walktrap", "Community_FastGreedy")]

item_communities <- item_communities[order(item_communities$Community_Louvain), ]

write.csv(item_communities, file.path(OUTPUT_DIR, "07_tables/09_item_communities.csv"), row.names = FALSE)

construct_communities <- data.frame(

Node = V(g_constructs)$name,

Community_Louvain = membership(louvain_constructs),

Community_Walktrap = membership(walktrap_constructs),

Community_FastGreedy = membership(fgreedy_constructs)

)

construct_communities$Display <- display_names[construct_communities$Node]

write.csv(construct_communities, file.path(OUTPUT_DIR, "07_tables/10_construct_communities.csv"), row.names = FALSE)

# 计算ARI

theoretical_communities <- data.frame(

Node = item_communities$Node,

Theoretical = as.numeric(factor(item_communities$Construct))

)

item_communities$Theoretical <- theoretical_communities$Theoretical[match(item_communities$Node, theoretical_communities$Node)]

if (requireNamespace("mclust", quietly = TRUE)) {

library(mclust)

ari_louvain <- adjustedRandIndex(item_communities$Theoretical, item_communities$Community_Louvain)

ari_walktrap <- adjustedRandIndex(item_communities$Theoretical, item_communities$Community_Walktrap)

ari_fgreedy <- adjustedRandIndex(item_communities$Theoretical, item_communities$Community_FastGreedy)

ari_summary <- data.frame(

Algorithm = c("Louvain", "Walktrap", "Fast-Greedy"),

ARI = round(c(ari_louvain, ari_walktrap, ari_fgreedy), 3)

)

cat("\n========== 与理论构念的一致性 (ARI) ==========\n")

print(ari_summary)

cat("==============================================\n\n")

write.csv(ari_summary, file.path(OUTPUT_DIR, "07_tables/11_ari_summary.csv"), row.names = FALSE)

} else {

cat("\n⚠️ mclust包未安装，跳过ARI计算。\n")

}

# ============================================================================

# 11.5.5 可视化社区发现结果（修复颜色）

# ============================================================================

# 条目层网络着色

png(file.path(OUTPUT_DIR, "01_networks/item_level/02_item_network_communities.png"),

width = 3500, height = 2500, res = 300)

comm_membership <- membership(louvain_items)

unique_comms <- sort(unique(comm_membership))

n_comms <- length(unique_comms)

if (n_comms <= 8) {

comm_palette <- brewer.pal(min(n_comms, 8), "Set1")

} else {

comm_palette <- rainbow(n_comms)

}

groups_list <- list()

for(i in 1:n_comms) {

comm_name <- paste("Community", i)

nodes_in_comm <- names(comm_membership[comm_membership == unique_comms[i]])

groups_list[[comm_name]] <- nodes_in_comm

}

qgraph(adj_items,

layout = "spring",

groups = groups_list,

color = comm_palette,

vsize = 6,

label.cex = 1.5,

label.scale = FALSE,

edge.width = 0.8,

minimum = 0.005,

cut = 0.05,

legend = TRUE,

legend.cex = 1.0,

legend.mode = "style2",

negDashed = TRUE,

title = "Item-Level Network (Louvain Communities)")

dev.off()

# 构念层网络着色

png(file.path(OUTPUT_DIR, "01_networks/construct_level/02_construct_network_communities.png"),

width = 3000, height = 2500, res = 300)

comm_membership_cons <- membership(louvain_constructs)

unique_comms_cons <- sort(unique(comm_membership_cons))

n_comms_cons <- length(unique_comms_cons)

if (n_comms_cons <= 8) {

comm_palette_cons <- brewer.pal(min(n_comms_cons, 8), "Set1")

} else {

comm_palette_cons <- rainbow(n_comms_cons)

}

groups_list_cons <- list()

for(i in 1:n_comms_cons) {

comm_name <- paste("Community", i)

nodes_in_comm <- names(comm_membership_cons[comm_membership_cons == unique_comms_cons[i]])

groups_list_cons[[comm_name]] <- nodes_in_comm

}

qgraph(adj_constructs,

layout = "circle",

groups = groups_list_cons,

color = comm_palette_cons,

vsize = 14,

label.cex = 1.8,

label.scale = FALSE,

labels = display_names,

edge.labels = FALSE,

legend = TRUE,

legend.cex = 1.0,

legend.mode = "style2",

title = "Construct-Level Network (Louvain Communities)")

dev.off()

cat("\n✅ 社区发现分析完成！\n")

cat(" - 生成文件:\n")

cat(" * 模块度表格: 08_modularity_summary.csv\n")

cat(" * 条目层社区归属: 09_item_communities.csv\n")

cat(" * 构念层社区归属: 10_construct_communities.csv\n")

if (exists("ari_summary")) {

cat(" * ARI一致性: 11_ari_summary.csv\n")

}

cat(" * 社区着色网络图: 02_item_network_communities.png, 02_construct_network_communities.png\n")

# ============================================================================

# 12. 稳定性分析

# ============================================================================

cat("[12] 正在进行稳定性分析 (1000次抽样，计算所有中心性指标的CS系数)...\n")

set.seed(2024)

cat(" - 构念层分析中...\n")

boot_construct <- bootnet(construct_data,

nBoots = 1000,

type = "case",

default = "EBICglasso",

tuning = 0.1,

statistics = c("strength", "closeness", "betweenness", "expectedInfluence"))

cs_construct_strength <- corStability(boot_construct, statistics = "strength")

cs_construct_closeness <- corStability(boot_construct, statistics = "closeness")

cs_construct_betweenness <- corStability(boot_construct, statistics = "betweenness")

cs_construct_expected <- corStability(boot_construct, statistics = "expectedInfluence")

cat(" - 条目层分析中（此步骤较耗时）...\n")

boot_item <- bootnet(item_data_complete,

nBoots = 1000,

type = "case",

default = "EBICglasso",

tuning = 0.1,

statistics = c("strength", "closeness", "betweenness", "expectedInfluence"))

cs_item_strength <- corStability(boot_item, statistics = "strength")

cs_item_closeness <- corStability(boot_item, statistics = "closeness")

cs_item_betweenness <- corStability(boot_item, statistics = "betweenness")

cs_item_expected <- corStability(boot_item, statistics = "expectedInfluence")

# ============================================================================

# 12.3 汇总CS系数表格

# ============================================================================

safe_get_cs <- function(var_name) {

if (exists(var_name)) {

val <- get(var_name)

if (length(val) == 1 && is.numeric(val) && !is.na(val)) {

return(val)

} else {

warning(paste0(var_name, " 存在但不是有效的单一数值（长度:", length(val), "），使用NA代替"))

return(NA)

}

} else {

warning(paste0(var_name, " 不存在，使用NA代替"))

return(NA)

}

}

cs_values <- c(

safe_get_cs("cs_construct_strength"),

safe_get_cs("cs_construct_closeness"),

safe_get_cs("cs_construct_betweenness"),

safe_get_cs("cs_construct_expected"),

safe_get_cs("cs_item_strength"),

safe_get_cs("cs_item_closeness"),

safe_get_cs("cs_item_betweenness"),

safe_get_cs("cs_item_expected")

)

if (any(is.na(cs_values))) {

cat("\n⚠️ 警告：以下CS系数缺失（将用NA代替）:\n")

all_names <- c("cs_construct_strength", "cs_construct_closeness", "cs_construct_betweenness",

"cs_construct_expected", "cs_item_strength", "cs_item_closeness",

"cs_item_betweenness", "cs_item_expected")

missing_names <- all_names[is.na(cs_values)]

for (name in missing_names) {

cat(" - ", name, "\n")

}

}

cs_summary <- data.frame(

Level = c(rep("Construct", 4), rep("Item", 4)),

Centrality_Index = rep(c("Strength", "Closeness", "Betweenness", "Expected Influence"), 2),

CS_Coefficient = round(cs_values, 3),

stringsAsFactors = FALSE

)

cs_summary$Interpretation <- ifelse(

is.na(cs_summary$CS_Coefficient),

"Not computed",

ifelse(cs_summary$CS_Coefficient >= 0.5, "Excellent (>= 0.5)",

ifelse(cs_summary$CS_Coefficient >= 0.25, "Good (>= 0.25)", "Poor (< 0.25)"))

)

cat("\n========== CS系数汇总表 ==========\n")

print(cs_summary)

cat("===================================\n\n")

write.csv(cs_summary, file.path(OUTPUT_DIR, "07_tables/07_cs_coefficients_all.csv"), row.names = FALSE)

save(boot_construct, boot_item, file = file.path(OUTPUT_DIR, "05_stability/bootstrap_objects.RData"))

png(file.path(OUTPUT_DIR, "05_stability/construct_stability_all_indicators.png"), width = 3000, height = 2400, res = 300)

plot(boot_construct, statistics = c("strength", "closeness", "betweenness", "expectedInfluence"), labels = display_names)

dev.off()

png(file.path(OUTPUT_DIR, "05_stability/item_stability_all_indicators.png"), width = 3200, height = 2800, res = 300)

plot(boot_item, statistics = c("strength", "closeness", "betweenness", "expectedInfluence"))

dev.off()

cat("\n========== CS系数汇总 ==========\n")

cat("构念层 - 强度 (Strength):", round(cs_construct_strength, 3),

ifelse(cs_construct_strength >= 0.5, "✓ 优秀", ifelse(cs_construct_strength >= 0.25, "✓ 良好", "✗ 不足")), "\n")

cat("构念层 - 紧密度 (Closeness):", round(cs_construct_closeness, 3),

ifelse(cs_construct_closeness >= 0.5, "✓ 优秀", ifelse(cs_construct_closeness >= 0.25, "✓ 良好", "✗ 不足")), "\n")

cat("构念层 - 中介度 (Betweenness):", round(cs_construct_betweenness, 3),

ifelse(cs_construct_betweenness >= 0.5, "✓ 优秀", ifelse(cs_construct_betweenness >= 0.25, "✓ 良好", "✗ 不足")), "\n")

cat("构念层 - 预期影响 (Expected Influence):", round(cs_construct_expected, 3),

ifelse(cs_construct_expected >= 0.5, "✓ 优秀", ifelse(cs_construct_expected >= 0.25, "✓ 良好", "✗ 不足")), "\n")

cat("条目层 - 强度 (Strength):", round(cs_item_strength, 3),

ifelse(cs_item_strength >= 0.5, "✓ 优秀", ifelse(cs_item_strength >= 0.25, "✓ 良好", "✗ 不足")), "\n")

cat("条目层 - 紧密度 (Closeness):", round(cs_item_closeness, 3),

ifelse(cs_item_closeness >= 0.5, "✓ 优秀", ifelse(cs_item_closeness >= 0.25, "✓ 良好", "✗ 不足")), "\n")

cat("条目层 - 中介度 (Betweenness):", round(cs_item_betweenness, 3),

ifelse(cs_item_betweenness >= 0.5, "✓ 优秀", ifelse(cs_item_betweenness >= 0.25, "✓ 良好", "✗ 不足")), "\n")

cat("条目层 - 预期影响 (Expected Influence):", round(cs_item_expected, 3),

ifelse(cs_item_expected >= 0.5, "✓ 优秀", ifelse(cs_item_expected >= 0.25, "✓ 良好", "✗ 不足")), "\n")

cat("================================\n")

# ============================================================================

# 13. 生成综合分析报告

# ============================================================================

cat("[13] 正在生成最终报告...\n")

if (!exists("cs_summary")) {

cs_summary <- data.frame(

Level = c("Construct", "Construct", "Construct", "Construct",

"Item", "Item", "Item", "Item"),

Centrality_Index = rep(c("Strength", "Closeness", "Betweenness", "Expected Influence"), 2),

CS_Coefficient = rep(NA, 8),

Interpretation = rep("Not computed", 8)

)

}

if (!exists("item_clustering")) {

item_clustering <- list(global = NA, mean_local = NA)

}

if (!exists("construct_clustering")) {

construct_clustering <- list(global = NA, mean_local = NA)

}

if (exists("cent_constructs_df") && nrow(cent_constructs_df) > 0) {

top_construct_node <- cent_constructs_df$Node[order(-cent_constructs_df$Strength)][1]

top_construct_strength <- max(cent_constructs_df$Strength, na.rm = TRUE)

top_construct_display <- ifelse(exists("display_names") && top_construct_node %in% names(display_names),

display_names[top_construct_node], top_construct_node)

} else {

top_construct_display <- "N/A"

top_construct_strength <- NA

}

if (exists("cent_items_df") && nrow(cent_items_df) > 0) {

top_item_row <- cent_items_df[order(-cent_items_df$Strength), ][1, ]

top_item_node <- top_item_row$Node

top_item_strength <- top_item_row$Strength

top_item_construct <- ifelse("Construct" %in% names(top_item_row), top_item_row$Construct, "Unknown")

} else {

top_item_node <- "N/A"

top_item_strength <- NA

top_item_construct <- "Unknown"

}

if (exists("bridge_df") && nrow(bridge_df) > 0) {

top_bridge_display <- bridge_df$Display[1]

top_bridge_strength <- bridge_df$Bridge_Strength[1]

} else {

top_bridge_display <- "N/A"

top_bridge_strength <- NA

}

item_density <- ifelse(exists("item_network"), round(item_network$density, 3), NA)

construct_density <- ifelse(exists("construct_network"), round(construct_network$density, 3), NA)

report_content <- c(

"# 心理网络分析综合报告",

paste("**分析时间:**", format(Sys.time(), "%Y-%m-%d %H:%M:%S")),

paste("**数据文件:**", basename(DATA_PATH)),

paste("**R脚本:** 02_psychological_network_analysis.R"),

"",

"## 1. 样本概况",

paste("- **总有效样本 (N):**", n),

paste("- **条目层网络密度:**", item_density),

paste("- **构念层网络密度:**", construct_density),

"",

"## 2. 核心网络特征",

"",

"### 2.1 聚类系数",

paste(" - **条目层**:"),

paste(" - 全局聚类系数 (WS):", round(item_clustering$global$WS, 3)),

paste(" - 全局聚类系数 (Barrat):", round(item_clustering$global$Barrat, 3)),

paste(" - 全局聚类系数 (Onnela):", round(item_clustering$global$Onnela, 3)),

paste(" - 全局聚类系数 (Zhang):", round(item_clustering$global$Zhang, 3)),

paste(" - 平均局部聚类系数 (WS):", round(item_clustering$mean_local_all$WS, 3)),

paste(" - 平均局部聚类系数 (Barrat):", round(item_clustering$mean_local_all$Barrat, 3)),

paste(" - 平均局部聚类系数 (Onnela):", round(item_clustering$mean_local_all$Onnela, 3)),

paste(" - 平均局部聚类系数 (Zhang):", round(item_clustering$mean_local_all$Zhang, 3)),

paste(" - **构念层**:"),

paste(" - 全局聚类系数 (WS):", round(construct_clustering$global$WS, 3)),

paste(" - 全局聚类系数 (Barrat):", round(construct_clustering$global$Barrat, 3)),

paste(" - 全局聚类系数 (Onnela):", round(construct_clustering$global$Onnela, 3)),

paste(" - 全局聚类系数 (Zhang):", round(construct_clustering$global$Zhang, 3)),

paste(" - 平均局部聚类系数 (WS):", round(construct_clustering$mean_local_all$WS, 3)),

paste(" - 平均局部聚类系数 (Barrat):", round(construct_clustering$mean_local_all$Barrat, 3)),

paste(" - 平均局部聚类系数 (Onnela):", round(construct_clustering$mean_local_all$Onnela, 3)),

paste(" - 平均局部聚类系数 (Zhang):", round(construct_clustering$mean_local_all$Zhang, 3)),

"",

"### 2.2 中心性识别 (基于Strength)",

paste(" - **构念层最强节点:**", top_construct_display,

"(强度 =", round(top_construct_strength, 3), ")"),

paste(" - **条目层最强节点:**", top_item_node,

"(强度 =", round(top_item_strength, 3), ", 所属构念:", top_item_construct, ")"),

"",

"### 2.3 最强连接边",

"#### 构念层 Top 5 连接:",

if (exists("top_constructs") && nrow(top_constructs) > 0) {

paste0(" - ", 1:min(5, nrow(top_constructs)), ". ",

display_names[top_constructs$from[1:min(5, nrow(top_constructs))]],

" — ",

display_names[top_constructs$to[1:min(5, nrow(top_constructs))]],

" (weight = ", top_constructs$weight[1:min(5, nrow(top_constructs))], ")")

} else {

" - 无可用数据"

},

"",

"#### 条目层 Top 5 连接:",

if (exists("top_items") && nrow(top_items) > 0) {

c(sapply(1:min(5, nrow(top_items)), function(i) {

paste0(" - ", i, ". ", top_items$from[i], " — ", top_items$to[i],

" (weight = ", top_items$weight[i], ")")

}))

} else {

" - 无可用数据"

},

"",

"## 3. 桥梁中心性分析",

paste("- **最强桥梁节点:**", top_bridge_display,

"(桥梁强度 =", round(top_bridge_strength, 3), ")"),

"",

"## 4. 网络稳定性分析 (CS系数)",

"",

"### 4.1 构念层 CS 系数 (Correlation Stability)",

if (exists("cs_summary")) {

cs_construct_rows <- cs_summary[cs_summary$Level == "Construct", ]

c(

paste(" - **强度 (Strength):**",

round(cs_construct_rows$CS_Coefficient[cs_construct_rows$Centrality_Index == "Strength"], 3),

"—", cs_construct_rows$Interpretation[cs_construct_rows$Centrality_Index == "Strength"]),

paste(" - **紧密度 (Closeness):**",

round(cs_construct_rows$CS_Coefficient[cs_construct_rows$Centrality_Index == "Closeness"], 3),

"—", cs_construct_rows$Interpretation[cs_construct_rows$Centrality_Index == "Closeness"]),

paste(" - **中介度 (Betweenness):**",

round(cs_construct_rows$CS_Coefficient[cs_construct_rows$Centrality_Index == "Betweenness"], 3),

"—", cs_construct_rows$Interpretation[cs_construct_rows$Centrality_Index == "Betweenness"]),

paste(" - **预期影响 (Expected Influence):**",

round(cs_construct_rows$CS_Coefficient[cs_construct_rows$Centrality_Index == "Expected Influence"], 3),

"—", cs_construct_rows$Interpretation[cs_construct_rows$Centrality_Index == "Expected Influence"])

)

} else {

" - CS系数数据不可用"

},

"",

"### 4.2 条目层 CS 系数",

if (exists("cs_summary")) {

cs_item_rows <- cs_summary[cs_summary$Level == "Item", ]

c(

paste(" - **强度 (Strength):**",

round(cs_item_rows$CS_Coefficient[cs_item_rows$Centrality_Index == "Strength"], 3),

"—", cs_item_rows$Interpretation[cs_item_rows$Centrality_Index == "Strength"]),

paste(" - **紧密度 (Closeness):**",

round(cs_item_rows$CS_Coefficient[cs_item_rows$Centrality_Index == "Closeness"], 3),

"—", cs_item_rows$Interpretation[cs_item_rows$Centrality_Index == "Closeness"]),

paste(" - **中介度 (Betweenness):**",

round(cs_item_rows$CS_Coefficient[cs_item_rows$Centrality_Index == "Betweenness"], 3),

"—", cs_item_rows$Interpretation[cs_item_rows$Centrality_Index == "Betweenness"]),

paste(" - **预期影响 (Expected Influence):**",

round(cs_item_rows$CS_Coefficient[cs_item_rows$Centrality_Index == "Expected Influence"], 3),

"—", cs_item_rows$Interpretation[cs_item_rows$Centrality_Index == "Expected Influence"])

)

} else {

" - CS系数数据不可用"

},

"",

"### 4.3 CS系数解释标准",

" - **≥ 0.50:** 优秀 (Excellent)",

" - **≥ 0.25:** 良好 (Good/Acceptable)",

" - **< 0.25:** 较差 (Poor)，解释需谨慎",

"",

"## 4.4 社区发现分析",

if (exists("modularity_summary")) {

mod_items <- modularity_items$Modularity[modularity_items$Algorithm == "Louvain"]

mod_constructs <- modularity_constructs$Modularity[modularity_constructs$Algorithm == "Louvain"]

n_items <- modularity_items$N_Communities[modularity_items$Algorithm == "Louvain"]

n_constructs <- modularity_constructs$N_Communities[modularity_constructs$Algorithm == "Louvain"]

c(

paste(" - **Louvain模块度 (条目层):**", round(mod_items, 3)),

paste(" - **Louvain模块度 (构念层):**", round(mod_constructs, 3)),

paste(" - **检测到的社区数量 (条目层):**", n_items),

paste(" - **检测到的社区数量 (构念层):**", n_constructs)

)

} else {

" - 社区发现数据不可用"

},

"",

"## 5. 输出文件汇总",

"### 5.1 网络图像",

" - `01_networks/construct_level/01_construct_network.png` - 构念层网络",

" - `01_networks/item_level/01_item_network_fixed.png` - 条目层网络",

" - `01_networks/combined_network.png` - 构念层与条目层对比图",

" - `01_networks/item_level/02_item_network_communities.png` - 条目层社区网络",

" - `01_networks/construct_level/02_construct_network_communities.png` - 构念层社区网络",

"",

"### 5.2 中心性图表",

" - `02_centrality/construct_level/01_construct_centrality.png` - 构念层中心性",

" - `02_centrality/item_level/01_item_centrality.png` - 条目层中心性",

"",

"### 5.3 稳定性分析",

" - `05_stability/construct_stability_all_indicators.png` - 构念层稳定性",

" - `05_stability/item_stability_all_indicators.png` - 条目层稳定性",

" - `05_stability/bootstrap_objects.RData` - Bootstrap原始对象",

"",

"### 5.4 数据表格",

" - `07_tables/01_item_descriptives.csv` - 条目层描述性统计",

" - `07_tables/01_construct_descriptives.csv` - 构念层描述性统计",

" - `07_tables/02_item_correlations.csv` - 条目层相关矩阵",

" - `07_tables/02_construct_correlations.csv` - 构念层相关矩阵",

" - `07_tables/03_item_centrality.csv` - 条目层中心性指标",

" - `07_tables/03_construct_centrality.csv` - 构念层中心性指标",

" - `07_tables/04_bridge_centrality.csv` - 桥梁中心性",

" - `07_tables/05_top_edges_construct.csv` - 构念层最强边",

" - `07_tables/06_top_edges_item.csv` - 条目层最强边",

" - `07_tables/07_cs_coefficients_all.csv` - 所有CS系数汇总",

" - `07_tables/08_modularity_summary.csv` - 模块度汇总",

" - `07_tables/09_item_communities.csv` - 条目层社区归属",

" - `07_tables/10_construct_communities.csv` - 构念层社区归属",

" - `07_tables/11_ari_summary.csv` - 与理论构念的一致性",

"",

"### 5.5 聚类分析",

" - `06_clustering/item_clustering_node_level.csv` - 条目层节点级聚类系数",

" - `06_clustering/construct_clustering_node_level.csv` - 构念层节点级聚类系数",

" - `06_clustering/item_clustering_global.csv` - 条目层全局聚类系数",

" - `06_clustering/construct_clustering_global.csv` - 构念层全局聚类系数",

" - `06_clustering/clustering_four_metrics_summary.csv` - 四指标汇总表",

" - `06_clustering/01_item_clustering_plot.png` - 条目层聚类图",

" - `06_clustering/01_construct_clustering_plot.png` - 构念层聚类图",

"",

"## 6. 研究备注",

paste("- **EBICglasso 调参:** gamma = 0.1"),

paste("- **Bootstrap 次数:** 1000"),

paste("- **缺失值处理:** 中位数填补"),

"",

"---",

paste("报告生成时间:", Sys.time()),

paste("** 本报告由 R 脚本自动生成 **")

)

writeLines(report_content, file.path(OUTPUT_DIR, "08_report/network_analysis_report.md"))

cat(" ✓ 综合报告已保存至:", file.path(OUTPUT_DIR, "08_report/network_analysis_report.md"), "\n")

txt_report <- c(

"=== 心理网络分析快速报告 ===",

paste("时间:", Sys.time()),

paste("样本量 N =", n),

paste("构念层密度 =", construct_density),

paste("条目层密度 =", item_density),

paste("最强桥梁节点:", top_bridge_display, "(", round(top_bridge_strength, 3), ")"),

"--- CS系数 (构念层) ---",

if (exists("cs_summary")) {

cs_construct_rows <- cs_summary[cs_summary$Level == "Construct", ]

c(

paste(" Strength:", round(cs_construct_rows$CS_Coefficient[cs_construct_rows$Centrality_Index == "Strength"], 3)),

paste(" Closeness:", round(cs_construct_rows$CS_Coefficient[cs_construct_rows$Centrality_Index == "Closeness"], 3)),

paste(" Betweenness:", round(cs_construct_rows$CS_Coefficient[cs_construct_rows$Centrality_Index == "Betweenness"], 3)),

paste(" Expected Influence:", round(cs_construct_rows$CS_Coefficient[cs_construct_rows$Centrality_Index == "Expected Influence"], 3))

)

} else {

" CS系数数据不可用"

},

"--- CS系数 (条目层) ---",

if (exists("cs_summary")) {

cs_item_rows <- cs_summary[cs_summary$Level == "Item", ]

c(

paste(" Strength:", round(cs_item_rows$CS_Coefficient[cs_item_rows$Centrality_Index == "Strength"], 3)),

paste(" Closeness:", round(cs_item_rows$CS_Coefficient[cs_item_rows$Centrality_Index == "Closeness"], 3)),

paste(" Betweenness:", round(cs_item_rows$CS_Coefficient[cs_item_rows$Centrality_Index == "Betweenness"], 3)),

paste(" Expected Influence:", round(cs_item_rows$CS_Coefficient[cs_item_rows$Centrality_Index == "Expected Influence"], 3))

)

} else {

" CS系数数据不可用"

}

)

writeLines(txt_report, file.path(OUTPUT_DIR, "08_report/quick_report.txt"))

cat(" ✓ 快速报告已保存至:", file.path(OUTPUT_DIR, "08_report/quick_report.txt"), "\n")

# ============================================================================

# 14. 保存所有结果对象

# ============================================================================

cat("[14] 正在保存所有数据对象...\n")

results_list <- list(

item_adj = adj_items,

construct_adj = adj_constructs,

item_centrality = cent_items_df,

construct_centrality = cent_constructs_df,

bridge_stats = bridge_df,

cs_summary = cs_summary,

item_clustering = item_clustering,

construct_clustering = construct_clustering,

top_edges_item = top_items,

top_edges_construct = top_constructs,

boot_construct = boot_construct,

boot_item = boot_item,

sample_size = n,

item_density = item_network$density,

construct_density = construct_network$density

)

save(results_list, file = file.path(OUTPUT_DIR, "network_analysis_final_results.RData"))

save.image(file.path(OUTPUT_DIR, "full_environment_backup.RData"))

cat("\n", rep("=", 70), "\n", sep = "")

cat("✅ 分析全部圆满完成！所有结果已保存。\n")

cat("请检查目录: ", OUTPUT_DIR, "\n")

cat(rep("=", 70), "\n\n")

## Part 4: 03_gender_comparison.R

# ============================================================================

# 03_gender_comparison.R

# 性别差异比较：心理网络分析（男生 vs 女生）

# 基于 02_Psychological_Network_Analysis.R 的整体结果

# 输出目录：output/03_Results

# ============================================================================

rm(list = ls())

cat("\n", rep("=", 70), "\n", sep = "")

cat("GENDER COMPARISON NETWORK ANALYSIS - FOR 13 SOCIAL SUPPORT STUDY\n")

cat(rep("=", 70), "\n\n")

# ============================================================================

# 1. 加载必要的包

# ============================================================================

cat("[1] 加载R包...\n")

required_packages <- c(

"qgraph", "bootnet", "networktools", "igraph", "ggplot2", "dplyr",

"tidyr", "psych", "RColorBrewer", "NetworkComparisonTest", "patchwork"

)

for (pkg in required_packages) {

if (!require(pkg, character.only = TRUE)) {

install.packages(pkg, dependencies = TRUE)

library(pkg, character.only = TRUE)

}

}

# ============================================================================

# 2. 路径设置

# ============================================================================

cat("[2] 设置工作路径与创建文件夹...\n")

BASE_DIR <- "XXX" # 请修改为你的实际路径

DATA_PATH <- "XXX" # 请修改为你的数据路径

OUTPUT_DIR <- file.path(BASE_DIR, "output/03_Results")

dir.create(OUTPUT_DIR, recursive = TRUE, showWarnings = FALSE)

GENDER_FIG_DIR <- file.path(OUTPUT_DIR, "figures")

GENDER_TABLES_DIR <- file.path(OUTPUT_DIR, "tables")

dir.create(GENDER_FIG_DIR, recursive = TRUE, showWarnings = FALSE)

dir.create(GENDER_TABLES_DIR, recursive = TRUE, showWarnings = FALSE)

# ============================================================================

# 3. 数据加载与预处理

# ============================================================================

cat("[3] 加载数据并分离性别...\n")

# 变量名（与02一致）

variable_names <- c(

"Grade", "Gender", "Age",

"G1", "G2", "G3", "G4",

"F1", "F2", "F3", "F4", "F5", "F6", "F7", "F8", "F9", "F10", "F11", "F12",

"S1", "S2", "S3", "S4", "S5", "S6", "S7", "S8",

"PA1", "PA2", "PA", "PS", "OS", "FS", "SE", "ZPA"

)

# 构念映射（与02完全一致）

construct_mapping <- list(

Parental_Support = list(

english = "Parental_Support",

display = "Parental Support",

items = c("F1", "F2", "F3", "F4"),

color = "#E69F00"

),

Peer_Support = list(

english = "Peer_Support",

display = "Peer Support",

items = c("F9", "F10", "F11", "F12"),

color = "#56B4E9"

),

Self_Efficacy = list(

english = "Self_Efficacy",

display = "Self-Efficacy",

items = paste0("S", 1:8),

color = "#009E73"

),

Football_Participation = list(

english = "Football_Participation",

display = "Football Participation",

items = c("PA1", "PA2"),

color = "#F0E442"

)

)

# 读取数据

raw_data <- read.csv(DATA_PATH, header = FALSE)

colnames(raw_data) <- variable_names[1:ncol(raw_data)]

raw_data[raw_data == 999] <- NA

# 提取所有分析条目（F1-F4, F9-F12, S1-S8, PA1-PA2）

all_items <- unlist(lapply(construct_mapping, function(x) x$items))

item_data <- raw_data[, all_items, drop = FALSE]

# 性别处理（假设1=男，2=女）

gender <- raw_data$Gender

cat(" 性别分布：\n")

print(table(gender, useNA = "ifany"))

# 按性别分割数据

boys_raw <- item_data[gender == 1, ]

girls_raw <- item_data[gender == 2, ]

# 缺失值处理：删除缺失过多的行（>30%缺失），然后用中位数填补

clean_gender_data <- function(df) {

missing_rate <- rowMeans(is.na(df))

df_clean <- df[missing_rate <= 0.3, ]

df_complete <- as.data.frame(lapply(df_clean, function(x) {

x[is.na(x)] <- median(x, na.rm = TRUE)

return(x)

}))

return(df_complete)

}

boys_data <- clean_gender_data(boys_raw)

girls_data <- clean_gender_data(girls_raw)

n_boys <- nrow(boys_data)

n_girls <- nrow(girls_data)

cat("\n 有效样本：男生 n =", n_boys, "；女生 n =", n_girls, "\n")

if (n_boys < 50 || n_girls < 50) {

warning("样本量较小（<50），NCT结果可能不稳定，请谨慎解释。")

}

# ============================================================================

# 4. 描述性统计与性别差异（Cohen's d）

# ============================================================================

cat("\n[4] 计算描述性统计及效应量...\n")

desc_stats <- function(data, group_name) {

means <- colMeans(data)

sds <- apply(data, 2, sd)

data.frame(Item = names(means), Group = group_name, Mean = means, SD = sds)

}

boys_desc <- desc_stats(boys_data, "Boys")

girls_desc <- desc_stats(girls_data, "Girls")

desc_wide <- merge(boys_desc, girls_desc, by = "Item", suffixes = c("_boys", "_girls"))

desc_wide <- desc_wide %>%

mutate(

pooled_sd = sqrt((SD_boys^2 + SD_girls^2) / 2),

cohen_d = (Mean_boys - Mean_girls) / pooled_sd,

d_abs = abs(cohen_d),

effect_size = case_when(

d_abs < 0.2 ~ "negligible",

d_abs < 0.5 ~ "small",

d_abs < 0.8 ~ "medium",

TRUE ~ "large"

)

) %>%

arrange(desc(d_abs))

write.csv(desc_wide, file.path(GENDER_TABLES_DIR, "01_descriptives_cohensd.csv"), row.names = FALSE)

cat(" 效应量最大的前5个条目：\n")

print(head(desc_wide[, c("Item", "Mean_boys", "Mean_girls", "cohen_d", "effect_size")], 5))

# ============================================================================

# 5. 分别估计男生和女生的网络（EBICglasso，gamma = 0.1）

# ============================================================================

cat("\n[5] 估计男生和女生网络...\n")

estimate_network <- function(data, group_name) {

cat(" 正在估计", group_name, "网络...\n")

cor_mat <- cor_auto(data) # 自动处理序数数据

adj <- EBICglasso(cor_mat, n = nrow(data), gamma = 0.1,

penalize.diagonal = FALSE, threshold = FALSE)

rownames(adj) <- colnames(adj) <- colnames(data)

# 计算网络统计量

n_edges <- sum(adj[upper.tri(adj)] != 0)

n_possible <- ncol(adj) * (ncol(adj) - 1) / 2

density <- n_edges / n_possible

pos_edges <- sum(adj > 0)

neg_edges <- sum(adj < 0)

mean_weight <- mean(adj[adj != 0])

cat(" 边数:", n_edges, "/", n_possible, "\n")

cat(" 密度:", round(density, 4), "\n")

cat(" 正边:", pos_edges, "负边:", neg_edges, "\n")

cat(" 平均权重:", round(mean_weight, 4), "\n")

return(list(adj = adj, stats = list(n_edges = n_edges, density = density, mean_weight = mean_weight)))

}

boys_net <- estimate_network(boys_data, "Boys")

girls_net <- estimate_network(girls_data, "Girls")

# 保存邻接矩阵

write.csv(round(boys_net$adj, 3), file.path(GENDER_TABLES_DIR, "02_adj_matrix_boys.csv"))

write.csv(round(girls_net$adj, 3), file.path(GENDER_TABLES_DIR, "02_adj_matrix_girls.csv"))

# ============================================================================

# 6. 网络可视化（并排比较）

# ============================================================================

cat("\n[6] 生成网络图...\n")

# 定义节点颜色（根据构念）

item_names <- colnames(boys_data)

get_node_color <- function(item) {

if (item %in% construct_mapping$Parental_Support$items) return(construct_mapping$Parental_Support$color)

if (item %in% construct_mapping$Peer_Support$items) return(construct_mapping$Peer_Support$color)

if (item %in% construct_mapping$Self_Efficacy$items) return(construct_mapping$Self_Efficacy$color)

if (item %in% construct_mapping$Football_Participation$items) return(construct_mapping$Football_Participation$color)

return("#CCCCCC")

}

node_colors <- sapply(item_names, get_node_color)

# 单独保存男生网络（可选：也调大标题字体）

png(file.path(GENDER_FIG_DIR, "01_network_boys.png"), width = 2500, height = 2000, res = 300)

qgraph(boys_net$adj, layout = "spring", labels = item_names, color = node_colors,

vsize = 5, label.cex = 1.2, edge.width = 0.8, negDashed = TRUE,

title = paste("Boys Network (n =", n_boys, ")"), title.cex = 1.8)

dev.off()

# 单独保存女生网络（可选：也调大标题字体）

png(file.path(GENDER_FIG_DIR, "01_network_girls.png"), width = 2500, height = 2000, res = 300)

qgraph(girls_net$adj, layout = "spring", labels = item_names, color = node_colors,

vsize = 5, label.cex = 1.2, edge.width = 0.8, negDashed = TRUE,

title = paste("Girls Network (n =", n_girls, ")"), title.cex = 1.8)

dev.off()

# 并排比较图（主要修改：添加 title.cex = 1.8 放大标题）

png(file.path(GENDER_FIG_DIR, "01_network_side_by_side.png"), width = 5000, height = 2200, res = 300)

par(mfrow = c(1, 2), mar = c(2, 2, 4, 2))

qgraph(boys_net$adj, layout = "spring", labels = item_names, color = node_colors,

vsize = 5, label.cex = 1.2, edge.width = 0.8, negDashed = TRUE,

title = paste("Boys (n =", n_boys, ")"), title.cex = 1.8, mar = c(5,5,5,5))

qgraph(girls_net$adj, layout = "spring", labels = item_names, color = node_colors,

vsize = 5, label.cex = 1.2, edge.width = 0.8, negDashed = TRUE,

title = paste("Girls (n =", n_girls, ")"), title.cex = 1.8, mar = c(5,5,5,5))

dev.off()

# ============================================================================

# 7. 中心性比较（强度、紧密度、中介性）

# ============================================================================

cat("\n[7] 计算中心性指标...\n")

calc_centrality <- function(adj, group) {

cent <- centrality(adj)

data.frame(

Item = colnames(adj),

Group = group,

Strength = cent$OutDegree,

Closeness = cent$Closeness,

Betweenness = cent$Betweenness,

ExpectedInfluence = cent$OutExpectedInfluence,

stringsAsFactors = FALSE

)

}

cent_boys <- calc_centrality(boys_net$adj, "Boys")

cent_girls <- calc_centrality(girls_net$adj, "Girls")

cent_combined <- merge(cent_boys, cent_girls, by = "Item", suffixes = c("_boys", "_girls"))

cent_combined <- cent_combined %>%

mutate(

Strength_diff = Strength_boys - Strength_girls,

Closeness_diff = Closeness_boys - Closeness_girls,

Betweenness_diff = Betweenness_boys - Betweenness_girls,

EI_diff = ExpectedInfluence_boys - ExpectedInfluence_girls

) %>%

arrange(desc(abs(Strength_diff)))

write.csv(cent_combined, file.path(GENDER_TABLES_DIR, "03_centrality_comparison.csv"), row.names = FALSE)

# 绘制中心性差异图

p_cent <- ggplot(cent_combined %>% slice_max(abs(Strength_diff), n = 10),

aes(x = reorder(Item, Strength_diff), y = Strength_diff, fill = Strength_diff > 0)) +

geom_col() + coord_flip() +

scale_fill_manual(values = c("TRUE" = "#56B4E9", "FALSE" = "#E69F00"),

labels = c("Higher in Girls", "Higher in Boys")) +

labs(title = "Strength Centrality Differences (Boys - Girls)",

x = "Item", y = "Difference in Strength") +

theme_minimal() + theme(legend.position = "bottom")

ggsave(file.path(GENDER_FIG_DIR, "02_centrality_diff.png"), p_cent, width = 10, height = 8, dpi = 300)

# ============================================================================

# 8. 网络比较检验（NCT）

# ============================================================================

cat("\n[8] 执行网络比较检验（NCT）...\n")

if (n_boys >= 30 && n_girls >= 30) { # NCT 对样本量有一定要求

set.seed(2024)

nct_result <- NCT(

data1 = boys_data,

data2 = girls_data,

it = 1000,

binary.data = FALSE,

paired = FALSE,

weighted = TRUE,

test.edges = TRUE,

edges = "all",

progressbar = TRUE,

test.centrality = TRUE,

centrality = c("strength", "closeness", "betweenness"),

p.adjust.methods = "holm"

)

# 保存 NCT 结果

save(nct_result, file = file.path(OUTPUT_DIR, "nct_results.RData"))

# 提取全局检验结果

nct_global <- data.frame(

Test = c("Global Strength Invariance", "Edge Weight Invariance"),

p_value = c(nct_result$glstrinv.pval, nct_result$nwinv.pval),

stringsAsFactors = FALSE

)

write.csv(nct_global, file.path(GENDER_TABLES_DIR, "04_nct_global.csv"), row.names = FALSE)

# 提取边差异（Holm校正后显著）

if (!is.null(nct_result$einv.pvals.adjusted)) {

edge_p_adj <- nct_result$einv.pvals.adjusted

sig_edges <- which(edge_p_adj < 0.05, arr.ind = TRUE)

if (length(sig_edges) > 0) {

sig_df <- data.frame(

Item1 = rownames(edge_p_adj)[sig_edges[,1]],

Item2 = colnames(edge_p_adj)[sig_edges[,2]],

p_adj = edge_p_adj[sig_edges]

)

sig_df <- sig_df[sig_df$Item1 != sig_df$Item2, ]

sig_df <- sig_df[!duplicated(t(apply(sig_df[,1:2], 1, sort))), ]

write.csv(sig_df, file.path(GENDER_TABLES_DIR, "04_nct_sig_edges.csv"), row.names = FALSE)

cat(" 显著边差异（Holm校正）:", nrow(sig_df), "\n")

} else {

cat(" 无显著边差异（Holm校正后）\n")

}

}

# 中心性差异检验

if (!is.null(nct_result$strength.pval)) {

cent_test <- data.frame(

Centrality = c("Strength", "Closeness", "Betweenness"),

p_value = c(nct_result$strength.pval, nct_result$closeness.pval, nct_result$betweenness.pval)

)

write.csv(cent_test, file.path(GENDER_TABLES_DIR, "04_nct_centrality_tests.csv"), row.names = FALSE)

}

cat(" NCT 完成。\n")

} else {

cat(" 样本量不足，跳过 NCT。\n")

}

# ============================================================================

# 9. 桥梁中心性分析（连接不同构念的节点）

# ============================================================================

cat("\n[9] 计算桥梁中心性...\n")

# 构建社区向量：每个条目所属构念的英文名

item_to_construct <- data.frame(

Item = all_items,

Construct = rep(names(construct_mapping), times = sapply(construct_mapping, function(x) length(x$items))),

stringsAsFactors = FALSE

)

# 确保顺序一致

item_to_construct <- item_to_construct[match(colnames(boys_data), item_to_construct$Item), ]

communities <- setNames(item_to_construct$Construct, item_to_construct$Item)

# 计算桥梁

bridge_boys <- bridge(boys_net$adj, communities = communities)

bridge_girls <- bridge(girls_net$adj, communities = communities)

# 整理成数据框

bridge_df_boys <- data.frame(

Item = names(bridge_boys$`Bridge Strength`),

BridgeStrength = bridge_boys$`Bridge Strength`,

BridgeBetweenness = bridge_boys$`Bridge Betweenness`,

BridgeCloseness = bridge_boys$`Bridge Closeness`,

Group = "Boys"

)

bridge_df_girls <- data.frame(

Item = names(bridge_girls$`Bridge Strength`),

BridgeStrength = bridge_girls$`Bridge Strength`,

BridgeBetweenness = bridge_girls$`Bridge Betweenness`,

BridgeCloseness = bridge_girls$`Bridge Closeness`,

Group = "Girls"

)

bridge_all <- rbind(bridge_df_boys, bridge_df_girls)

write.csv(bridge_all, file.path(GENDER_TABLES_DIR, "05_bridge_centrality.csv"), row.names = FALSE)

# 绘制桥梁强度对比

bridge_wide <- merge(

bridge_df_boys[, c("Item", "BridgeStrength")],

bridge_df_girls[, c("Item", "BridgeStrength")],

by = "Item", suffixes = c("_boys", "_girls")

) %>% mutate(diff = BridgeStrength_boys - BridgeStrength_girls)

p_bridge <- ggplot(bridge_wide %>% slice_max(abs(diff), n = 10),

aes(x = reorder(Item, diff), y = diff, fill = diff > 0)) +

geom_col() + coord_flip() +

scale_fill_manual(values = c("TRUE" = "#56B4E9", "FALSE" = "#E69F00"),

labels = c("Higher in Girls", "Higher in Boys")) +

labs(title = "Bridge Strength Differences (Boys - Girls)",

x = "Item", y = "Difference in Bridge Strength") +

theme_minimal() + theme(legend.position = "bottom")

ggsave(file.path(GENDER_FIG_DIR, "03_bridge_diff.png"), p_bridge, width = 10, height = 8, dpi = 300)

# ============================================================================

# 10. 构念内部连接性分析

# ============================================================================

cat("\n[10] 分析各构念内部连接性...\n")

construct_internal <- function(adj, construct_items) {

idx <- which(colnames(adj) %in% construct_items)

if (length(idx) < 2) return(NA)

sub_adj <- adj[idx, idx]

upper <- sub_adj[upper.tri(sub_adj)]

mean_weight <- mean(abs(upper))

density <- sum(upper != 0) / length(upper)

c(mean_weight = mean_weight, density = density)

}

constructs <- names(construct_mapping)

internal_res <- data.frame(Construct = constructs)

for (con in constructs) {

items <- construct_mapping[[con]]$items

boys_stat <- construct_internal(boys_net$adj, items)

girls_stat <- construct_internal(girls_net$adj, items)

internal_res[internal_res$Construct == con, "Boys_mean_weight"] <- boys_stat["mean_weight"]

internal_res[internal_res$Construct == con, "Boys_density"] <- boys_stat["density"]

internal_res[internal_res$Construct == con, "Girls_mean_weight"] <- girls_stat["mean_weight"]

internal_res[internal_res$Construct == con, "Girls_density"] <- girls_stat["density"]

}

write.csv(internal_res, file.path(GENDER_TABLES_DIR, "06_construct_internal.csv"), row.names = FALSE)

# ============================================================================

# 11. 保存完整工作空间与报告生成

# ============================================================================

cat("\n[11] 保存所有结果并生成报告...\n")

# 保存工作空间

save.image(file.path(OUTPUT_DIR, "gender_analysis_complete.RData"))

# 生成文本报告

sink(file.path(OUTPUT_DIR, "gender_analysis_report.txt"))

cat("性别差异网络分析报告\n")

cat("====================\n\n")

cat("分析时间：", as.character(Sys.time()), "\n")

cat("男生样本量：", n_boys, "\n")

cat("女生样本量：", n_girls, "\n\n")

cat("网络密度：男生", round(boys_net$stats$density, 4), "；女生", round(girls_net$stats$density, 4), "\n")

cat("平均边权：男生", round(boys_net$stats$mean_weight, 4), "；女生", round(girls_net$stats$mean_weight, 4), "\n\n")

cat("效应量最大的5个条目（Cohen's d）：\n")

print(head(desc_wide[, c("Item", "cohen_d", "effect_size")], 5))

cat("\n")

cat("中心性差异最大的5个条目（强度）：\n")

top5 <- cent_combined %>% slice_max(abs(Strength_diff), n = 5) %>% select(Item, Strength_boys, Strength_girls, Strength_diff)

print(top5)

cat("\n")

if (exists("nct_result")) {

cat("网络比较检验（NCT）：\n")

cat(" 全局强度不变性 p =", nct_result$glstrinv.pval, "\n")

cat(" 边权重不变性 p =", nct_result$nwinv.pval, "\n")

if (exists("sig_df") && nrow(sig_df) > 0) {

cat(" 显著边差异（Holm校正）数量：", nrow(sig_df), "\n")

}

}

cat("\n所有表格和图形已保存至：", OUTPUT_DIR, "\n")

sink()

cat("\n✅ 性别差异分析完成！结果保存在：", OUTPUT_DIR, "\n")
